# Ellipticity and the problem of iterates in Denjoy–Carleman classes

**DOI:** 10.1007/s13348-024-00455-7

**Published:** 2024-09-28

**Authors:** Stefan Fürdös, Gerhard Schindl

**Affiliations:** 1 Instituto de Matemática e Estatística, Universidade de São Paulo, Rua do Matão 1010, São Paulo, SP 05508-090 Brazil; 2 Present Address: Fakultät für Mathematik, Universität Wien, Oskar-Morgenstern-Platz 1, 1090 Wien, Austria

**Keywords:** Problem of iterates, Ultradifferentiable vectors, Strong non-quasianalyticity, Non-elliptic operators, Optimal functions in Denjoy–Carleman classes, Primary 35B65, Secondary 26E10, 35J99, 46E10

## Abstract

In 1978 Métivier showed that a linear differential operator *P* with analytic coefficients is elliptic if and only if the theorem of iterates holds for *P* with respect to any non-analytic Gevrey class. In this paper we extend this theorem to Denjoy–Carleman classes given by strongly non-quasianalytic weight sequences. The proof involves a new way to construct optimal functions in Denjoy–Carleman classes via Fourier integrals, which might be of independent interest. Moreover, we point out that the analogous statement for Braun–Meise–Taylor classes given by weight functions cannot hold. This signifies an important difference in the properties of Denjoy–Carleman classes and Braun–Meise–Taylor classes, respectively.

## Introduction

In this paper we continue our investigation of the problem of iterates using the modern theory of ultradifferentiable classes which was started in [20]. There has been recently a lot of work regarding the problem of iterates in various settings, e.g. [3, 4, 11, 13–18] and [38]. For a survey of the older literature we refer to [8].

In a simple form the problem of iterates is the following question: If some derivatives of a smooth function *u* satisfy certain uniform estimates, does it follow that all derivatives of *u* satisfy these estimates? The first fundamental result in this direction appeared in the literature when in 1962 it was proven by Kotake-Narasimhan [30] and Komatsu [28] separately that if *P* is an elliptic differential operator of order *d* with analytic coefficients in some open set $\Omega \subseteq {\mathbb {R}}^n$ then a smooth function $u\in {\mathcal {E}}(\Omega )$ is analytic in $\Omega$ if for all compact subsets *K* of $\Omega$ there is a constant $C>0$ such that

1.1 $$\begin{aligned} \Vert {P^k u}\Vert _{L^2(K)}\le C^{k+1} (dk)! \end{aligned}$$

for all non-negative integers $k\in {\mathbb {N}}_0$. Similar results have been obtained by Nelson [33] for elliptic systems of analytic vector fields. Functions, which satisfy (1.1), are usually referred to as analytic vectors of the operator *P*.

Generally, given an ultradifferentiable structure^1^${\mathcal {U}}$ we say that a smooth function *u* is an ultradifferentiable vector of class ${\mathcal {U}}$ (or short: a ${\mathcal {U}}$-vector) of the operator *P* if the iterates $P^ku$ satisfy the defining estimates of ${\mathcal {U}}$. The problem of iterates for a given operator is then the study of the regularity of ultradifferentiable vectors of this operator. In various different settings it has been proven that, if the operator *P* is elliptic, then the theorem of iterates for *P* holds, i.e. every ultradifferentiable vector of *P* is an ultradifferentiable function of the same class, see e.g. [4, 5, 7, 11, 14] and [21].

On the other hand, the problem of iterates for non-elliptic operators was for example considered in [1, 6, 18] and [20]. In particular, Métivier [32] showed that for any non-elliptic differential operator *P* with analytic coefficients and any non-analytic Gevrey class ${\mathcal {G}}^s$, i.e. $s>1$, there is an *s*-Gevrey vector of *P* which is not an *s*-Gevrey function. The question, which one can now pose, is the following: Can we extend this result to more general classes?

In the literature there are mainly two families of ultradifferentiable classes which generalize the family of Gevrey classes: First, the Denjoy–Carleman classes given by weight sequences, see [29] and, secondly, the Braun–Meise–Taylor classes given by weight functions, in the modern form introduced in [12]. We may note that these classes do not necessarily coincide according to [9].

In [20, Section 5D] the authors extended Métivier’s theorem to Denjoy–Carleman classes given by the sequences $(q^{k^2})_k$, where $q>1$ is a parameter. On the other hand, for Braun–Meise–Taylor classes given by certain non-quasianalytic weight functions Juan-Huguet [27] showed that for operators with constant coefficients the theorem of iterates holds if and only if the operator is elliptic. However, [20, Theorem 1.1] states that the theorem of iterates holds for hypoelliptic operators of principal type with analytic coefficients with respect to Braun–Meise–Taylor classes given by a certain subfamiliy of weight functions.

In this article, we will prove that Métivier’s Theorem holds for every strongly non-quasianalytic weight sequence. Using the Borel map we can give an invariant version of our result. Recall that the Borel map $\lambda _0$ takes every smooth function to the sequence of its derivatives evaluated at the origin. Clearly the image of any ultradifferentiable structure ${\mathcal {U}}$ is contained in the space $\Lambda _{{\mathcal {U}}}$ of sequences which satisfy analogous estimates. We call $\lambda _0:\, {\mathcal {U}}\rightarrow \Lambda _{{\mathcal {U}}}$ the Borel map associated to the structure ${\mathcal {U}}$. Applying Petzsche’s work [34] on the Borel map associated to Denjoy–Carleman classes we obtain the following version of our main theorem:

### Theorem 1.1

If the Borel map associated to the Denjoy–Carleman class ${\mathcal {E}}^{[{\textbf{M}}]}$ is surjective then for any non-elliptic differential operator *P* with analytic coefficients there is an ultradifferentiable vector *u* of class $[{\textbf{M}}]$ of *P* which is not an element of ${\mathcal {E}}^{[{\textbf{M}}]}$.

For the notations and definitions used above see Sect. 2 and for the precise context of Theorem 1.1 we refer in particular to Remark 2.7. Moreover, we need to point out that the analogous statement for Braun–Meise–Taylor classes is not true. In fact, if we consider the subfamily of weight functions from [20, Theorem 1.1] mentioned above, then we observe that the Borel map associated to the Braun–Meise–Taylor classes defined by these weight functions is surjective, see Remark 2.8 for more details. This shows a significant difference between Denjoy–Carleman classes and Braun–Meise–Taylor classes regarding the problem of iterates.

The proof of our main theorem is a modification of the arguments in [32]. In particular, the vector *u* in the assertion of Theorem 1.1 is constructed as a Fourier integral, which is a smooth function but there is a weight sequence ${\textbf{N}}$, depending only on ${\textbf{M}}$ and *P* such that ${\mathcal {E}}^{\{{\textbf{N}}\}}$ is strictly larger than ${\mathcal {E}}^{\{{\textbf{M}}\}}$ and *u* is not an element in any Denjoy–Carleman class strictly smaller than ${\mathcal {E}}^{\{{\textbf{N}}\}}$. Moreover, if the original class ${\mathcal {E}}^{\{{\textbf{M}}\}}$ is closed under derivation we can show that *u* is indeed an element of ${\mathcal {E}}^{\{{\textbf{N}}\}}$, i.e. *u* is an optimal function of the class ${\mathcal {E}}^{\{{\textbf{N}}\}}$. Historically, the construction of optimal functions in Denjoy–Carleman classes was mainly done by using Fourier series, see e.g. [37]. We believe that this new approach using integrals to construct optimal functions will have various applications. It uses an integral kernel, which is very closely related to the so-called $\{{\textbf{N}}\}$–optimal flat functions introduced in [23], which are holomorphic functions defined in a sector of the Riemann surface of the logarithm whose asymptotic expansion at the origin is the zero series and the rate of convergence is exactly determined by the weight sequence ${\textbf{N}}$. We should also note that the existence of such optimal $\{{\textbf{N}}\}$–flat functions, see [23], is closely related to the non-vanishing of an invariant $\gamma ({\textbf{N}})$ of the weight sequence ${\textbf{N}}$ which was first introduced by Thilliez [36]. This invariant itself will also play a significant role in the proof of our main theorem.

The paper is structured in the following way: We present the fundamental definitions of Denjoy–Carleman classes and the precise formulation of our main results in Sect. 2. In Sect. 3 we assemble the technical results we need for the proof of these statements. The proofs themselves are contained in Sect. 4. In the last Sect. 5 we discuss the optimality of the functions constructed in Sect. 4.

## Statement of main results

In this note $\Omega$ will always denote an open subset of the Euclidean space ${\mathbb {R}}^n$, ${\mathbb {N}}$ is the set of positive integers and ${\mathbb {N}}_0={\mathbb {N}}\cup \{0\}$.

### Definition 2.1

In our setting a weight sequence is a sequence ${\textbf{M}}=(M_k)_{k\in {\mathbb {N}}_0}$ of positive numbers such that $M_0=1\le M_1$ and the sequence $\mu _k=M_k/M_{k-1}$ is increasing and satisfies $\lim _{k\rightarrow \infty }\mu _k=\infty$.

### Remark 2.2

The fact that the sequence $(\mu _k)_{k\in {\mathbb {N}}}$ is increasing is equivalent to the logarithmic convexity of the weight sequence $(M_k)_k$, i.e.

2.1 $$\begin{aligned} M_k^2\le M_{k-1}M_{k+1} \end{aligned}$$

for all $k\in {\mathbb {N}}$. Then it follows that the sequence $\root k \of {M_k}$ is increasing and

2.2 $$\begin{aligned} \lim _{k\rightarrow \infty }\root k \of {M_k}=\infty . \end{aligned}$$

If *K* is a compact subset of ${\mathbb {R}}^n$ then ${\mathcal {E}}(K)$ is the space of smooth functions *f* for which there is a neighborhood $U_f$ of *K* such that $f\in {\mathcal {E}}(U_f)$.

If ${\textbf{M}}$ is a weight sequence then we can define two spaces of ultradifferentiable functions on *K*: First, a smooth function $f\in {\mathcal {E}}(K)$ is an element of the Roumieu class ${\mathcal {E}}^{\{ {\textbf{M}} \}} ( K )$ associated to ${\textbf{M}}$ if there are constants $C,h>0$ such that

2.3 $$\begin{aligned} \sup _{x\in K}\,{|} D^\alpha f(x) {|}\le Ch^{|\alpha |}M_{|\alpha |}\end{aligned}$$

for all $\alpha \in {\mathbb {N}}_0^n$. On the other hand a function $u\in {\mathcal {E}}(K)$ is an element of the Beurling class ${\mathcal {E}}^{( {\textbf{M}} )} ( K )$ if for all $h>0$ there is a constant $C>0$ such that (2.3) holds for all $\alpha \in {\mathbb {N}}_0^n$. We will use the notation $[{\textbf{M}}]=\{{\textbf{M}}\},({\textbf{M}})$ throughout the paper to refer simultaneously to the Roumieu and Beurling case.

If $\Omega \subseteq {\mathbb {R}}^n$ is an open set then the local classes ${\mathcal {E}}^{[ {\textbf{M}} ]} ( \Omega )$ are given in the following way: A smooth function $f\in {\mathcal {E}}(\Omega )$ is an element of ${\mathcal {E}}^{[ {\textbf{M}} ]} ( \Omega )$ if for all compact subsets *K* of $\Omega$ we have that $f\vert _K\in {\mathcal {E}}^{[ {\textbf{M}} ]} ( K )$. For more details on the functional analytic structure of these spaces we refer to [29].

If *P* is a differential operator of order *d* with coefficients in ${\mathcal {E}}^{\{ {\textbf{M}} \}} ( \Omega )$ for some weight sequence ${\textbf{M}}$ then we say that a distribution $u\in {\mathcal {D}}^\prime (\Omega )$ is an ultradifferentiable Roumieu vector associated to ${\textbf{M}}$ of *P* (or *u* is an $\{{\textbf{M}}\}$-vector of *P*) if $P^ku\in L^2_{loc}(\Omega )$ for all $k\in {\mathbb {N}}_0$ (we use here the convention $P^0={{\,\textrm{Id}\,}}$) and for all compact $K\subseteq \Omega$ there are constants $C,h>0$ such that

2.4 $$\begin{aligned} \Vert {P^ku}\Vert _{L^2(K)}\le Ch^k M_{dk} \end{aligned}$$

for all $k\in {\mathbb {N}}_0$. On the other hand, if the coefficients of *P* are in ${\mathcal {E}}^{( {\textbf{M}} )} ( \Omega )$ then the distribution *u* is an ultradifferentiable Beurling vector associated to ${\textbf{M}}$ of *P* (short: $({\textbf{M}})$-vector of *P*) if $P^ku\in L^2_{loc}(\Omega )$ for every $k\in {\mathbb {N}}_0$ and for all compact $K\subseteq$ $\Omega$ and all $h>0$ there is some constant $C>0$ such that (2.4) is satisfied for all $k\in {\mathbb {N}}_0$. The space of all $[{\textbf{M}}]$-vectors of *P* is denoted by ${\mathcal {E}}^{[ {\textbf{M}} ]} ( \Omega ; P )$. We have always ${\mathcal {E}}^{[ {\textbf{M}} ]} ( \Omega )\subseteq {\mathcal {E}}^{[ {\textbf{M}} ]} ( \Omega ; P )$, cf. [19]. If the weight sequence ${\textbf{M}}$ satisfies

2.5 $$\begin{aligned} \lim _{k\rightarrow \infty } \root k \of {\frac{M_k}{k!}}=\infty , \end{aligned}$$

then ${\mathcal {E}}^{[ {\textbf{M}} ]} ( \Omega )\subseteq {\mathcal {E}}^{[ {\textbf{M}} ]} ( \Omega ; P )$ for all operators *P* with analytic coefficients, since condition (2.5) means in particular that the space of analytic functions ${\mathcal {A}}(\Omega )$ is (strictly) contained in ${\mathcal {E}}^{[ {\textbf{M}} ]} ( \Omega )$.

If *P* is an elliptic differential operator with analytic coefficients and the weight sequence ${\textbf{M}}$ satisfies both (2.5) and

2.6 $$\begin{aligned} \sup _{k\in {\mathbb {N}}}\root k \of {\frac{M_{k}}{M_{k-1}}}<\infty \end{aligned}$$

then we have that the theorem of iterates holds, i.e. ${\mathcal {E}}^{[ {\textbf{M}} ]} ( \Omega ; P )={\mathcal {E}}^{[ {\textbf{M}} ]} ( \Omega )$. The Roumieu case was proven in [7], for the Beurling case see [20]. We may note that (2.6) implies that ${\mathcal {E}}^{[ {\textbf{M}} ]} ( \Omega )$ is closed under derivation.

Our main theorem gives a converse to this statement. We say a weight sequence ${\textbf{M}}$ is strongly non-quasianalytic if there is a constant *A* such that

2.7 $$\begin{aligned} \sum _{k=j+1}^\infty \frac{M_{k-1}}{M_k}\le A(j+1)\frac{M_j}{M_{j+1}} \end{aligned}$$

for all $$j\in {\mathbb {N}}_0$$.

### Theorem 2.3

Let *P* be a non-elliptic differential operator with analytic coefficients in some open set $\Omega \subseteq {\mathbb {R}}^n$. If ${\textbf{M}}$ is a strongly non-quasianalytic weight sequence then there is a smooth function $u\in {\mathcal {E}}(\Omega )$ such that

$$\begin{aligned} u\in {\mathcal {E}}^{ ({\textbf{M}}) } ( \Omega ; P )\!\setminus \!{\mathcal {E}}^{\{ {\textbf{M}} \}} ( \Omega ). \end{aligned}$$

### Remark 2.4

We may point out that the proof of Theorem 2.3 gives actually a slightly stronger result: There is a weight sequence ${\widetilde{{\textbf{M}}}}$ depending on ${\textbf{M}}$ and *P* such that $u\in {\mathcal {E}}^{\{ {\widetilde{{\textbf{M}}}} \}} \left( \Omega ; P \right) \subseteq {\mathcal {E}}^{ ({\textbf{M}}) } ( \Omega ; P )$.

Since (2.7) implies (2.5) we obtain immediately as a corollary of Theorem 2.3 a generalization of [32, Theorem 1.2]:

### Corollary 2.5

Let *P* be a differential operator with analytic coefficients in $\Omega$ and ${\textbf{M}}$ be a strongly non-quasianalytic weight sequence which also satisfies (2.6). Then the following statements are equivalent.

1. The operator *P* is elliptic in $\Omega$.
2. $${\mathcal {E}}^{\{ {\textbf{M}} \}} \left( \Omega ; P \right) ={\mathcal {E}}^{\{ {\textbf{M}} \}} ( \Omega )$$.
3. $${\mathcal {E}}^{ ({\textbf{M}}) } ( \Omega ; P )={\mathcal {E}}^{( {\textbf{M}} )} ( \Omega )$$.

### Example 2.6

1. Let $s\ge 1$. The Gevrey sequence ${\textbf{G}}^s=((k!)^s)_k$ is a strongly non-quasianalytic weight sequence if $s>1$. Furthermore it is easy to see that ${\textbf{G}}^s$ satisfies (2.6) for all $s\ge 1$. We will denote the Gevrey class of order *s* by ${\mathcal {G}}^s(\Omega )={\mathcal {E}}^{\{ {\textbf{G}}^s \}} ( \Omega )$.
2. Let $q>1$ and $r>1$. The weight sequence ${\textbf{N}}^{q,r}$ given by $N^{q,r}_k=q^{k^r}$ is strongly non-quasianalytic for every $q>1$ and $r>1$ but (2.6) holds if and only if $1<r\le 2$.
3. Let $\sigma > 0$. The weight sequence ${\textbf{L}}^\sigma$ given by $L^\sigma _k=k!(\log (e+k))^{\sigma k}$ is not strongly non-quasianalytic but satisfies (2.5) and (2.6) for every $\sigma >0$.

### Remark 2.7

Let $\Lambda$ be the space of all complex sequences $a=(a_j)_j$ and if ${\textbf{M}}$ is a weight sequence then let us denote by $\Lambda _{\{{\textbf{M}}\}}$ the space of all complex sequences $a\in \Lambda$ for which there are constants $C,h>0$ such that

2.8 $$\begin{aligned} {|} a_j {|}\le Ch^jM_j \end{aligned}$$

for all $j\in {\mathbb {N}}_0$. The set $\Lambda _{({\textbf{M}})}$ consists of all $a\in \Lambda$ such that for every $h>0$ there is some $C>0$ so that (2.8) holds for every $j\in {\mathbb {N}}_0$.

The Borel map $\lambda _0:\,{\mathcal {E}}([-1,1])\rightarrow \Lambda$ at the origin is defined by

$$\begin{aligned} \lambda _0(f):=\left( f^{(j)}(0)\right) _{j\in {\mathbb {N}}_0},\qquad f\in {\mathcal {E}}([-1,1]). \end{aligned}$$

Clearly, $\lambda _0({\mathcal {E}}^{[ {\textbf{M}} ]} ( [-1,1] ))\subseteq \Lambda _{[{\textbf{M}}]}$. According to [34] the restricted Borel map

$$\begin{aligned} \lambda _0\vert _{{\mathcal {E}}^{[ {\textbf{M}} ]} ( [-1,1] )}:{\mathcal {E}}^{[ {\textbf{M}} ]} ( [-1,1] )\longrightarrow \Lambda _{[{\textbf{M}}]} \end{aligned}$$

is surjective if and only if the weight sequence ${\textbf{M}}$ is strongly non-quasianalytic in both the Roumieu and Beurling cases. Combining this fact with Theorem 2.3 gives instantly Theorem 1.1.

### Remark 2.8

1. As we have mentioned above, ellipticity of the operators considered implies that the theorem of iterates holds in a lot of different ultradifferentiable structures, see e.g. [20]. If we turn our attention to Braun–Meise–Taylor classes ${\mathcal {E}}^{[ \omega ]} ( \Omega )$ given by weight functions $\omega$ then we know that ${\mathcal {E}}^{[ \omega ]} ( \Omega ; P )={\mathcal {E}}^{[ \omega ]} ( \Omega )$ for all elliptic differential operators with analytic coefficients in $\Omega$ if $\omega (t)=o(t)$, c.f. [20, Corollary 3.19], see also [3] (for a definition of ${\mathcal {E}}^{[ \omega ]} ( \Omega )$ and ${\mathcal {E}}^{[ \omega ]} ( \Omega ; P )$ we refer e.g. to [20]). On the other hand Juan-Huguet [27] gave a statement which might be considered as an variant of Corollary 2.5: If *Q* is a differential operator with constant coefficients then [27, Theorem 4.12] states that for a non-quasianalytic^2^ weight function $\omega$ with
  2.9 $$\begin{aligned} \,\,\exists \,H\ge 1:\qquad 2\omega (t)\le \omega (Ht)+H,\qquad t>0, \end{aligned}$$
  we have ${\mathcal {E}}^{[ \omega ]} ( \Omega ; Q )={\mathcal {E}}^{[ \omega ]} ( \Omega )$ if and only if *Q* is elliptic. In fact the theorem of Juan-Huguet is a complement to Corollary 2.5. Namely, according to [9] condition (2.9) implies that there is a weight sequence ${\textbf{N}}$ such that ${\mathcal {E}}^{[ {\textbf{N}} ]} ( \Omega )={\mathcal {E}}^{[ \omega ]} ( \Omega )$ and ${\mathcal {E}}^{[ {\textbf{N}} ]} ( \Omega ; P )={\mathcal {E}}^{[ \omega ]} ( \Omega ; P )$. Moreover, there is some $s>1$ such that ${\mathcal {E}}^{[ \omega ]} ( \Omega )\subseteq {\mathcal {G}}^s(\Omega )$, this follows from [9, 14 Theorem & 16 Corollary] combined with [31, Proposition 2.6]. On the other hand, for every strongly non-quasianalytic weight sequence ${\textbf{M}}$ there is some $\sigma >1$ such that ${\mathcal {G}}^{\sigma }(\Omega )\subseteq {\mathcal {E}}^{[ {\textbf{M}} ]} ( \Omega )$ by [36, Section 1.3].
2. It remains the question, if there are analogous versions of Theorem 2.3, resp.  Theorem 1.1 (and thus also Corollary 2.5) for Braun–Meise–Taylor classes. Or formulated differently: If the Borel map associated to ${\mathcal {E}}^{[\omega ]}$ is surjective and we have ${\mathcal {E}}^{[ \omega ]} ( \Omega ; P )={\mathcal {E}}^{[ \omega ]} ( \Omega )$ for a differential operator *P* with analytic coefficients in $\Omega$, can we conclude that *P* is elliptic? The answer to this question is negative. First, observe that according to [10] the Borel map associated to ${\mathcal {E}}^{[\omega ]}$ is surjective if and only if $\omega$ satisfies the strong non-quasianalyticity condition for weight functions:
  2.10 $$\begin{aligned} \int _1^\infty \!\frac{\omega (ty)}{t^2}\,dt =O(\omega (y))\qquad \text {for } y\rightarrow \infty . \end{aligned}$$
  On the other hand, [20, Theorem 1.1] states that for each hypoelliptic differential operator *P* of principal type with analytic coefficients we have ${\mathcal {E}}^{[ \omega ]} ( \Omega ; P )={\mathcal {E}}^{[ \omega ]} ( \Omega )$ if the weight function $\omega$ satisfies
  2.11 $$\begin{aligned} \,\,\exists \,H>0:\quad \omega \bigl (t^2\bigr )=O(\omega (Ht)) \qquad \text {for } t\rightarrow \infty . \end{aligned}$$
  It follows from [26, Lemma A.1] and [25, Lemma 4.3] that condition (2.11) implies property (2.10). Thence the analogue of Theorem 1.1 in the category of Braun–Meise–Taylor classes cannot hold.
3. We may note that [20, Theorem 1.1] is consistent with [27, Theorem 4.12]: If a weight function $\omega$ satisfies (2.11) then ${\mathcal {G}}^r(\Omega )\subseteq {\mathcal {E}}^{\{ \omega \}} ( \Omega )$ for all $r>1$: By [35, Corollary 5.17] it is enough to show that $\omega (t)=o(t^{1/s})$ for all $s>1$. Now, if $\omega$ satisfies (2.11) then for all $\gamma >1$ there exists $C>0$ such that
  $$\begin{aligned} \omega (t^\gamma )\le C(\omega (t)+1) \end{aligned}$$
  by [20, Lemma 5.4]. Thus it follows that $\omega (t^\gamma )$ is non-quasianalytic, i.e.
  $$\begin{aligned} \int _1^\infty \frac{\omega (t^\gamma )}{t^2}\,dt<\infty . \end{aligned}$$
  Therefore $\omega (t^\gamma )=o(t)$ for $\gamma >1$ arbitrary. Moreover, in [20] it was shown that if $\omega$ is a weight function satisfying (2.11) then ${\mathcal {E}}^{[ \omega ]} ( \Omega )$ cannot be described as a Denjoy–Carleman class given by some weight sequence.

Finally we note that in the proof of Theorem 2.3 the assumption that the coefficients of the operator *P* are analytic is not really necessary. In fact, we only need that the coefficients are regular enough relative to the weight sequence ${\textbf{M}}$. However, the analyticity of the coefficients allows us to write down our main Theorem 2.3 in a concise form. Nevertheless, in the case of Gevrey classes we give another generalization of [32, Theorem 1.2]:

### Theorem 2.9

Let $1\le r<s$. If *P* is a differential operator with coefficients in ${\mathcal {G}}^r(\Omega )$ for some open set $\Omega \subseteq {\mathbb {R}}^n$ then the following statements are equivalent:

1. *P* is elliptic in $\Omega$.
2. $${\mathcal {G}}^s(\Omega ,P)={\mathcal {G}}^s(\Omega )$$.

## Preliminaries

### Weight sequences

In this Section we summarize the facts on weight sequences and the associated spaces which we will need for the proof of Theorem 2.3. We begin by recalling an auxiliary result from [20, Section 6D]:

#### Lemma 3.1

( [20, Lemma 6.10]) Let ${\textbf{M}}$ be a weight sequence and $\rho , R\ge 1$. Then

$$\begin{aligned} \rho ^jM_{k+l}R^l\le \rho ^{j+l}M_k+M_{j+k+l}R^{j+l} \end{aligned}$$

for all $$j,k,l\in {\mathbb {N}}_0$$.

#### Proof

For $\rho \ge \mu _{k+l}R$ we obtain that

$$\begin{aligned} M_{k+l}R^l=M_k\mu _{k+1}R\dots \mu _{k+l}R\le \rho ^{l}M_k \end{aligned}$$

since $\mu _k$ is increasing. If $\rho \le \mu _{k+l}R$ then

$$\begin{aligned} \rho ^j\le \mu _{k+l+1}R\dots \mu _{k+l+j}R\le \frac{M_{j+k+l}}{M_{k+l}}R^j. \end{aligned}$$

$$\square $$

We define on the set of all weight sequences the following order relations: Let ${\textbf{M}}$ and ${\textbf{N}}$ be two weight sequences then

- ${\textbf{M}}\le A{\textbf{N}}$ for some $A>0$ if $M_k\le A N_k$ for all $k\in {\mathbb {N}}_0$.
- ${\textbf{M}}\preceq {\textbf{N}}$ if there are constants $C,h>0$ such that $M_k\le Ch^k N_k$ for all $k\in {\mathbb {N}}_0$.
- ${\textbf{M}}\lhd {\textbf{N}}$ if for every $h>0$ there is a constant $C>0$ such that $M_k\le Ch^kN_k$ for all $k\in {\mathbb {N}}_0$.

Moreover, we write ${\textbf{M}}\approx {\textbf{N}}$ if ${\textbf{M}}\preceq {\textbf{N}}$ and ${\textbf{N}}\preceq {\textbf{M}}$. It is clear that ${\textbf{M}}\preceq {\textbf{N}}$ implies that ${\mathcal {E}}^{[ {\textbf{M}} ]} ( \Omega )\subseteq {\mathcal {E}}^{[ {\textbf{N}} ]} ( \Omega )$ and ${\mathcal {E}}^{[ {\textbf{M}} ]} ( \Omega ; P )\subseteq {\mathcal {E}}^{[ {\textbf{N}} ]} ( \Omega ; P )$ for any differential operator *P* of class $[{\textbf{M}}]$. If ${\textbf{M}}\lhd {\textbf{N}}$ then ${\mathcal {E}}^{\{ {\textbf{M}} \}} ( \Omega )\subseteq {\mathcal {E}}^{( {\textbf{N}} )} ( \Omega )$ and ${\mathcal {E}}^{\{ {\textbf{M}} \}} \left( \Omega ; P \right) \subseteq {\mathcal {E}}^{ ({\textbf{N}}) } ( \Omega ; P )$. We may occasionally need the following condition: We say that a weight sequence ${\textbf{M}}$ is strongly logarithmic convex if

3.1 $$\begin{aligned} \left( \frac{M_k}{k!}\right) ^2\le \frac{M_{k-1}}{(k-1)!}\frac{M_{k+1}}{(k+1)!},\qquad \,\,\forall \,k\in {\mathbb {N}}. \end{aligned}$$

#### Lemma 3.2

Let ${\textbf{M}}$ be a strongly logarithmic convex weight sequence. Then we have that

$$\begin{aligned} \left( {\begin{array}{c}k\\ j\end{array}}\right) M_{k-j}M_{j+\ell }\le M_{k+\ell }, \end{aligned}$$

for all $j,k,\ell \in {\mathbb {N}}_0$ with $j\le k$.

#### Proof

If ${\textbf{M}}$ is strongly logarithmic convex, i.e. (3.1) holds, and $M_0/0!=M_0=1$ then it is well known that

$$\begin{aligned} \frac{M_j}{j!}\frac{M_k}{k!}\le \frac{M_{j+k}}{(j+k)!} \end{aligned}$$

for all $j,k\in {\mathbb {N}}_0$. This proves the assertion for $\ell =0$.

In the case $\ell \ge 1$ we observe that

$$\begin{aligned} \begin{aligned} \left( {\begin{array}{c}k\\ j\end{array}}\right) M_{k-j}M_{j+\ell }&= \frac{k!(j+\ell )!}{j!} \frac{M_{k-j}}{(k-j)!} \frac{M_{j+\ell }}{(j+\ell )!}\\&\le k!\left( \prod _{\nu =1}^\ell (j+\nu )\right) \frac{M_{k+\ell }}{(k+\ell )!}\\&\le k!\left( \prod _{\nu =1}^\ell (k+\nu )\right) \frac{M_{k+\ell }}{(k+\ell )!}\\&= M_{k+\ell }. \end{aligned} \end{aligned}$$

$$\square $$

#### Remark 3.3

If ${\textbf{M}}$ is a strongly non-quasianalytic weight sequence, then there is a strongly logarithmic weight sequence ${\widetilde{{\textbf{M}}}}$ such that ${\textbf{M}}\approx {\widetilde{{\textbf{M}}}}$, by [34, 1.1 Proposition (a)].

There is another concept which we need to introduce. We denote the space of smooth functions with compact support in $\Omega$ by ${\mathcal {D}}(\Omega )$.

#### Definition 3.4

A subspace *E* of ${\mathcal {E}}(\Omega )$ is called quasianalytic if $E\cap {\mathcal {D}}(\Omega )=\{0\}$, i.e. *E* contains no non-trivial functions with compact support.

In the case of Denjoy–Carleman classes the Denjoy–Carleman theorem characterizes the quasianalyticity of the spaces, see e.g. [22] or [29]:

#### Theorem 3.5

Let ${\textbf{M}}$ be a weight sequence. Then the class ${\mathcal {E}}^{[ {\textbf{M}} ]} ( \Omega )$ is not quasianalytic if and only if

3.2 $$\begin{aligned} \sum _{k=0}^\infty \frac{M_k}{M_{k+1}}<\infty . \end{aligned}$$

We say that a weight sequence ${\textbf{M}}$ is non-quasianalytic if (3.2) is satisfied and quasianalytic otherwise. Clearly (2.7) implies (3.2).

### Associated weights

#### Definition 3.6

If ${\textbf{M}}$ is a weight sequence then

$$\begin{aligned} \omega _{\textbf{M}}(t)&=\sup _{k\in {\mathbb {N}}_0}\log \frac{t^k}{M_k},\quad t>0,&\omega _{\textbf{M}}(0)&=0, \end{aligned}$$

is the weight function associated to ${\textbf{M}}$. The function

$$\begin{aligned} h_{\textbf{M}}(t)&=\inf _{k\in {\mathbb {N}}_0}t^kM_k,\qquad t>0,&h_{\textbf{M}}(0)&=0, \end{aligned}$$

is the weight associated to ${\textbf{M}}$.

It is clear that $\omega _{\textbf{M}}$ and $h_{\textbf{M}}$ are well-defined, continuous and increasing functions on the right half-line $[0,\infty )$ due to (2.1) and (2.2). Moreover it is easy to see that

3.3 $$\begin{aligned} h_{\textbf{M}}\left( t^{-1}\right) =e^{-\omega _{\textbf{M}}(t)} \end{aligned}$$

for $t>0$. We can recover the weight sequence from its associated weight function:

3.4 $$\begin{aligned} M_k=\sup _{t\ge 0}\frac{t^k}{\exp (\omega _{\textbf{M}}(t))},\qquad k\in {\mathbb {N}}_0. \end{aligned}$$

To any pair $({\textbf{M}},{\textbf{N}})$ of weight sequences we can define the pointwise product ${\textbf{M}}{\textbf{N}}={\textbf{M}}\cdot {\textbf{N}}$ by

$$\begin{aligned} (MN)_k=M_kN_k,\qquad k\in {\mathbb {N}}_0. \end{aligned}$$

If $\tau >0$ then we define similarly the power ${\textbf{M}}^\tau$ of the weight sequence ${\textbf{M}}$ by $M_k^\tau =(M_k)^\tau$. Both ${\textbf{M}}{\textbf{N}}$ and ${\textbf{M}}^\tau$ are again weight sequences. We will need the following result.

#### Lemma 3.7

Let ${\textbf{T}}$ and ${\textbf{U}}$ be two weight sequences and $\tau >1$. Then the following two assertions are equivalent:

1. There is a constant $A\ge 1$ such that
  $$\begin{aligned} {\textbf{U}}\le A{\textbf{T}}^\tau . \end{aligned}$$
2. There is a constant $C\ge 1$ such that
  $$\begin{aligned} \omega _{{\textbf{T}}}(s)\le \tau ^{-1}\omega _{{\textbf{U}}}\bigl (s^\tau \bigr )+C \end{aligned}$$
  for all $$s\ge 0$$.

If one of the above conditions holds for ${\textbf{T}}$, ${\textbf{U}}$ and $\tau$ then for all $0<a<1$, and $\sigma >\tau$ there exists a constant $C\ge 1$ such that

3.5 $$\begin{aligned} \omega _{{\textbf{T}}}(s)\le \tau ^{-1}\omega _{{\textbf{U}}}\bigl (as^\sigma \bigr )+C \end{aligned}$$

for all $$s\ge 0$$.

#### Proof

We begin by assuming (2). Since both ${\textbf{T}}$ and ${\textbf{U}}$ are weight sequences we can apply (3.4) and obtain for all $k\in {\mathbb {N}}_0$ that

$$\begin{aligned} T_k&=\sup _{s\ge 0}\frac{s^k}{\exp (\omega _{{\textbf{T}}}(s))}\ge \frac{1}{e^C} \sup _{s\ge 0}\frac{s^k}{\exp (\tau ^{-1}\omega _{{\textbf{U}}}(s^{\tau }))} =\frac{1}{e^C}\sup _{t\ge 0}\frac{t^{k/\tau }}{\exp (\tau ^{-1}\omega _{{\textbf{U}}}(t))} \\  &=\frac{1}{e^C}\left( \sup _{t\ge 0}\frac{t^{k}}{\exp (\omega _{{\textbf{U}}}(t))}\right) ^{1/\tau } =\frac{1}{e^C}(U_k)^{1/\tau }, \end{aligned}$$

thus

$$\begin{aligned} U_k\le e^{C\tau }(T_k)^{\tau } \end{aligned}$$

for all $k\in {\mathbb {N}}_0$ and (1) is verified with $A:=e^{C\tau }$.

If (1) holds then let us first recall the following immediate consequence of taking any power $a>0$ of a given weight sequence ${\textbf{M}}$:

$$\begin{aligned} \omega _{{\textbf{M}}^a}(t)=\sup _{k\in {\mathbb {N}}_0}\log \left( \frac{t^{k/a}}{M_k}\right) ^a =a\omega _{{\textbf{M}}}\left( t^{1/a}\right) \end{aligned}$$

for all $t\ge 0$. By assumption we have $t^k/(T_k)^{\tau }\le At^k/U_k$ for all $t\ge 0$ and $k\in {\mathbb {N}}_0$ and thus by the above and the definition of associated weight functions we get for all $t\ge 0$ that

$$\begin{aligned} \tau \omega _{{\textbf{T}}}(t^{1/\tau }) =\omega _{{\textbf{T}}^{\tau }}(t)\le \omega _{{\textbf{U}}}(t)+\log (A). \end{aligned}$$

Thus (2) is verified with the same $\tau$ and $C:=\log A/\tau$.

In order to prove the last statement assume that (2) holds and note that for all $\sigma >\tau$ we have $s^{\tau }\le as^{\sigma }$ for all *s* sufficiently large (depending on given $a<1$ and $1<\tau <\sigma$). Thus (3.5) is verified if we sufficiently enlarge the constant *C*. $\square$

### Asymptotic expansions

Let ${\mathcal {R}}$ be the Riemann surface of the logarithm and ${\mathbb {C}}[[z]]$ be the space of formal power series with complex coefficients. We denote the space of holomorphic functions defined on an open subset *U* of ${\mathcal {R}}$ by ${\mathcal {O}}(U)$. If *S* is a sector of ${\mathcal {R}}$ we say that a holomorphic function $g\in {\mathcal {O}}(S)$ admits ${\hat{g}}=\sum _j a_jz^j\in {\mathbb {C}}[[z]]$ as its uniform $\{{\textbf{M}}\}$-asymptotic expansion if there are constants $C,A>0$ such that for all $k\in {\mathbb {N}}_0$ we have

$$\begin{aligned} {|} f(z)-\sum _{j=0}^{k-1}a_jz^j {|}<CA^kM_k{|} z {|}^k,\quad z\in S. \end{aligned}$$

We denote the space of holomorphic functions in *S* admitting an uniform $\{{\textbf{M}}\}$-asymptotic expansion by $\widetilde{{\mathcal {O}}}^{\{{\textbf{M}}\}}(S)$.

We are here mainly interested in holomorphic functions defined on unbounded sectors bisected by the real line, i.e. sectors of the form

$$\begin{aligned} S_\gamma =\left\{ z\in {\mathcal {R}}: {|} \arg z {|}<\frac{\gamma \pi }{2} \right\} \end{aligned}$$

with opening $\gamma \pi$ where $\gamma >0$.

#### Definition 3.8

( [23, Definition 3.3]) Let ${\textbf{M}}$ be a weight sequence and *S* be an unbounded sector in ${\mathcal {R}}$ bisected by the interval $(0,\infty )$. A holomorphic function $G\in {\mathcal {O}}(S)$ is called an optimal $\{{\textbf{M}}\}$-flat function in *S* if

1. There are constants $A_1$ and $B_1$ such that
  3.6 $$\begin{aligned} A_1h_{\textbf{M}}\left( B_1t\right) \le G(t) \end{aligned}$$
  for all $$t>0$$.
2. There exist $A_2,B_2>0$ such that
  3.7 $$\begin{aligned} {|} G(z) {|}\le A_2h_{\textbf{M}}\bigl (B_2{|} z {|}\bigr ) \end{aligned}$$
  for all $$z\in S$$.

If *G* is an optimal $\{{\textbf{M}}\}$-flat function in a sector *S* then condition (1) in particular gives that $G(t)>0$ for $t>0$. Moreover, we derive from (3.7) that

$$\begin{aligned} {|} G(z) {|}\le A_2B_2^k M_k{|} z {|}^k, \qquad k\in {\mathbb {N}}_0,\; z\in S, \end{aligned}$$

which implies that $G\in \widetilde{{\mathcal {O}}}^{\{{\textbf{M}}\}}(S)$ with the null series being the uniform asymptotic expansion of *G* in *S*. On the other hand, the estimate (3.6) gives that the rate of decrease on the real line to 0 is precisely controlled by the sequence ${\textbf{M}}$. This motivates the terminology.

If *G* is an optimal $\{{\textbf{M}}\}$-flat function in *S* then we introduce the kernel function $\Phi =\Phi _{\textbf{M}}:\, S\rightarrow {\mathbb {C}}$ defined by

$$\begin{aligned} \Phi (z)=G\left( \frac{1}{z}\right) . \end{aligned}$$

Thus we have the following inequalities:

$$\begin{aligned} A_1 e^{-\omega _{\textbf{M}}(t/B_1)}=A_1h_{\textbf{M}}\left( \frac{B_1}{t}\right) \le \Phi (t) \end{aligned}$$

for $$t>0$$ and

3.8 $$\begin{aligned} {|} \Phi (z) {|}\le A_2h_{\textbf{M}}\left( \frac{B_2}{{|} z {|}}\right) =A_2e^{-\omega _{\textbf{M}}({|} z {|}/B_2)} \end{aligned}$$

for $z\in S$. The following Proposition is essential for the proof of Theorem 2.3.

#### Proposition 3.9

(cf.  [23, Proposition 3.11]) Let ${\textbf{M}}$ be a weight sequence and $G\in \widetilde{{\mathcal {O}}}^{\{{\textbf{M}}\}}(S)$ be an optimal $\{{\textbf{M}}\}$-flat function in a sector $S\subseteq {\mathcal {R}}$, which is bisected by the real line $(0,\infty )$. If we put $\Phi (z)=G(1/z)$ then there is a constant $Q_1>0$ such that

$$\begin{aligned} Q_1^{k+1}M_k\le \int _0^\infty \! t^k\Phi (t)\,dt \end{aligned}$$

for all $$k\in {\mathbb {N}}_0$$.

Moreover, if ${\textbf{M}}$ satisfies (2.6) then there exists some $Q_2>0$ such that

$$\begin{aligned} \int _0^\infty \! t^k\Phi (t)\,dt\le Q^{k+1}_2M_k \end{aligned}$$

for all $$k\in {\mathbb {N}}_0$$.

Proposition 3.9 follows directly from the proof of [23, Proposition 3.11].

Finally we need to discuss conditions on the weight sequence ${\textbf{M}}$ which ensure the existence of optimal $\{{\textbf{M}}\}$-flat functions. It turns out to be useful to consider a growth index associated to weight sequences, which was introduced by Thilliez [36] while investigating the surjectivity of the asymptotic Borel map. We will use here the characterization given in [24]. We recall that a sequence ${\textbf{L}}=(L_k)_k$ is almost increasing if there is some constant $C>0$ such that

$$\begin{aligned} L_j\le CL_k \end{aligned}$$

for all $$j\le k$$.

#### Definition 3.10

If ${\textbf{M}}$ is a weight sequence then we define the index $\gamma ({\textbf{M}})$ of ${\textbf{M}}$ by

$$\begin{aligned} \gamma ({\textbf{M}})=\sup \left\{ \gamma >0: \text {The sequence } \biggl (\frac{\mu _k}{k^\gamma }\biggr )_k \text {is almost increasing} \right\} \in [0,\infty ]. \end{aligned}$$

It follows directly from the definition that $\gamma ({\textbf{G}}^s)=s$ for $s\ge 1$ and $\gamma ({\textbf{N}}^{q,r})=\infty$ for all $q,r>1$. More generally, it is easy to see that if ${\textbf{M}}$ is a weight sequence and $\tau >0$ then

$$\begin{aligned} \gamma \bigl ({\textbf{M}}^\tau \bigr )=\tau \gamma ({\textbf{M}}). \end{aligned}$$

We may also note that a weight sequence ${\textbf{M}}$ is strongly non-quasianalytic if and only if $\gamma ({\textbf{M}})>1$, see [24]. Finally, according to [23, Proposition 3.10] the following statement is true: If ${\textbf{M}}$ is a weight sequence such that $\gamma ({\textbf{M}})>0$ then for each $0<\gamma <\gamma ({\textbf{M}})$ there exists an optimal $\{{\textbf{M}}\}$-flat function *G* in the sector $S_\gamma$.

## Proof of the main theorem

In order to prove Theorem 2.3 we will try to adapt the pattern of the proof of [32, Theorem 2.3]. So let

$$\begin{aligned} P=P(x,D)=\sum _{{|\alpha |}\le d} p_\alpha (x)D^\alpha , \quad p_\alpha \in {\mathcal {E}}(\Omega ), \end{aligned}$$

be a differential operator of order *d* with smooth coefficients in an open set $\Omega \subseteq {\mathbb {R}}^n$. The symbol of *P* is denoted by

$$\begin{aligned} p(x,\xi )&=\sum _{{|\alpha |}\le d} p_\alpha (x)\xi ^\alpha ,\qquad x\in \Omega ,\;\xi \in {\mathbb {R}}^n\!\setminus \!\{0\}, \end{aligned}$$

whereas

$$\begin{aligned} p_d(x,\xi )&=\sum _{{|\alpha |}= d} p_\alpha (x)\xi ^\alpha ,\qquad x\in \Omega ,\;\xi \in {\mathbb {R}}^n\!\setminus \!\{0\}, \end{aligned}$$

is the principal symbol of *P*. If we suppose that *P* is not elliptic then there has to be a point $x_0\in \Omega$ and a unit vector $\xi _0\in S^{n-1}=\left\{ \xi \in {\mathbb {R}}^n: {|\xi |}=1 \right\}$ such that $p_d(x_0,\xi _0)=0$. Let $\delta >0$ be such that $B_0=\left\{ x\in {\mathbb {R}}^n: {|} x-x_0 {|}\le 2\delta \right\} \subseteq \Omega$. Then we have the following Lemma.

### Lemma 4.1

There is a constant $D>0$ such that for all $t>1$, $0<\varepsilon <1$ and all $x\in \Omega$ with ${|} x-x_0 {|}\le 2\delta t^{-\varepsilon }$ we have the estimate

4.1 $$\begin{aligned} {|} p(x, t\xi _0) {|}\le Dt^{d-\varepsilon }. \end{aligned}$$

### Proof

Since $t>1$ and $\varepsilon >0$ we have that

$$\begin{aligned} B_t=\left\{ x\in {\mathbb {R}}^n: {|} x-x_0 {|}\le 2\delta t^{-\varepsilon } \right\} \subseteq B_0. \end{aligned}$$

We are going to write

$$\begin{aligned} p_j(x,\xi )=\sum _{{|\alpha |}=j}p_\alpha (x)\xi ^\alpha \end{aligned}$$

for the homogeneous part of order $j\in \left\{ 0,\dotsc ,d-1 \right\}$ of *p* and estimate

$$\begin{aligned} {|} p_j(x,t\xi _0) {|}\le t^{j}\sup _{x\in B_0}{|} p_j(x,\xi _0) {|} \le t^{d-\varepsilon }\sup _{\begin{array}{c} x\in B_0\\ j=0,1,\dotsc ,d-1 \end{array}}{|} p_j(x,\xi _0) {|} \end{aligned}$$

for $x\in B_t$, $t>1$ and $0<\varepsilon <1$. In the case of the principal symbol $p_d$ we have that

$$\begin{aligned} p_d(x,t\xi _0)=t^dp_d(x,\xi _0). \end{aligned}$$

Since $p_d(x_0,\xi _0)=0$ it follows from [2, Lemma 1.2] that there are continuous functions $g_k$, $k=1,\dotsc ,n$, in $B_0$ such that

$$\begin{aligned} p_d(x,\xi _0)=\sum _{k=1}^n (x-x_0)^{e_k}g_k(x) \end{aligned}$$

where $e_k$ denotes the *k*-th unit vector. If $x\in B_t$, $t>1$ and $0<\varepsilon <1$ we can thus estimate

$$\begin{aligned} \begin{aligned} {|} p_d(x,t\xi _0) {|}&\le t^d {|} x-x_0 {|} \sum _{k=1}^n{|} g_k(x) {|}\\&\le 2\delta t^{d-\varepsilon }n\sup _{\begin{array}{c} x\in B_0\\ k=1,\dotsc ,n \end{array}}{|} g_k(x) {|}. \end{aligned} \end{aligned}$$

$$\square $$

After this technical observation we begin with the construction of the $\{{\textbf{M}}\}$-vector *u* for a given weight sequence ${\textbf{M}}$. We may suppose that there is a non-quasianalytic weight sequence ${\textbf{L}}$ such that ${\textbf{L}}\preceq {\textbf{M}}$. Then there exists $\psi \in {\mathcal {E}}^{\{ {\textbf{L}} \}} ( {\mathbb {R}}^n )$ such that

4.2 $$ \begin{aligned} \psi (x)=1\quad \text {for}\; {|} x {|}<\delta \qquad { \& }\qquad \psi (x)=0\quad \text {for}\; {|} x {|}>2\delta . \end{aligned}$$

We define the function *u* by

4.3 $$\begin{aligned} u(x)=\int _1^\infty \negthickspace \psi \left( t^\varepsilon (x-x_0)\right) \Phi _{\textbf{N}}(t)e^{it\xi _0(x-x_0)}\,dt,\qquad x\in \Omega , \end{aligned}$$

where $0<\varepsilon <1$ is a parameter to be specified and $\Phi _{\textbf{N}}(t)=G_{\textbf{N}}(1/t)$ with $G_{\textbf{N}}$ being an optimal $\{{\textbf{N}}\}$-flat function for some weight sequence ${\textbf{N}}$. Obviously $u\in {\mathcal {E}}(\Omega )$ with ${{\,\textrm{supp}\,}}u\subseteq B_{0}$ and we observe that if $D_{\xi _0}=-i\partial _{\xi _0}$ then

$$\begin{aligned} D_{\xi _0}^k u(x_0)=\int _{1}^{\infty }\negthickspace t^{k}\Phi _{\textbf{N}}(t)\,dt. \end{aligned}$$

Since $h_{\textbf{N}}(s)\le 1$ for all $s>0$ we have that

$$\begin{aligned} \begin{aligned} 0< \int _0^1\!t^k \Phi _{\textbf{N}}(t)\,dt\le C\int _0^1\!t^k\,dt= \frac{C}{k+1} \xrightarrow {\;k\rightarrow \infty \;} 0 \end{aligned} \end{aligned}$$

for some constant $C>0$. Hence using Proposition 3.9 we can conclude that there is a constant $Q_1>0$ such that

4.4 $$\begin{aligned} Q_1^{k+1} N_k-\frac{C}{k+1}\le {|} D_{\xi _0}^ku(x_0) {|} \end{aligned}$$

for all $k\in {\mathbb {N}}_0$. It follows that *u* cannot be of class $\{{\textbf{M}}\}$ in any neighborhood of $x_0$ if ${\textbf{M}}\precnapprox {\textbf{N}}$.

In order to estimate $P^ku$ we need to assume some a-priori regularity on the coefficients of *P*. Therefore we will suppose that $p_\alpha \in {\mathcal {E}}^{\{ {\textbf{L}} \}} ( \Omega )$ for all $\alpha$, which means that there is a constant $C_P>0$ such that for all $\nu \in {\mathbb {N}}_0^n$, all $\alpha \in {\mathbb {N}}_0^n$ with ${|\alpha |}\le d$, all $x\in B_0$ and every $t\ge 1$ we have that

4.5 $$\begin{aligned} {|} D^\nu _x \partial _\xi ^\alpha p\bigl (x,t\xi _0\bigr ) {|} \le C_P^{|\nu |+1}L_{|\nu |} t^{d-{|\alpha |}}. \end{aligned}$$

Now, if we compute $P^ku$, $k\in {\mathbb {N}}_0$, we see that

4.6 $$\begin{aligned} P^ku(x)=\int _1^\infty \negthickspace Q_k(x,t)\Phi _{\textbf{N}}(t)e^{i\xi _0(x-x_0)}\,dt \end{aligned}$$

where the functions $Q_k$ are iteratively defined by

4.7a $$\begin{aligned} Q_0(x,t)&= \psi \bigl (t^{\varepsilon }(x-x_0)\bigr ) \end{aligned}$$

and

4.7b $$\begin{aligned} Q_{k+1}(x,t)&=\sum _{{|\alpha |}\le d}\frac{1}{\alpha !}\partial _\xi ^\alpha p(x,t\xi _0) D^\alpha _x Q_k(x,t),\qquad k\in {\mathbb {N}}_0. \end{aligned}$$

We will need the following Lemma:

### Lemma 4.2

If ${\textbf{L}}$ is a strongly non-quasianalytic weight sequence then there exist constants $A>0$, $C_0>0$ and $h_0>0$ such that for all $k\in {\mathbb {N}}_0$, all $\nu \in {\mathbb {N}}_0^n$, all $x\in B_{0}$ and all $t\ge 1$ the following estimate holds:

4.8 $$\begin{aligned} {|} D_x^\nu Q_k(x,t) {|}\le C_0\left( h_0t^\varepsilon \right) ^{|\nu |}A ^k \left( t^{(d-\varepsilon )k}L_{|\nu |}+t^{k\varepsilon (2d-1)}L_{|\nu |+dk}\right) . \end{aligned}$$

### Proof

According to Remark 3.3 we can assume without loss of generality that ${\textbf{L}}$ is strongly logarithmic convex. Thus we still have (4.5) with a possibly different constant $C_P$. Since $\psi \in {\mathcal {D}}^{\{{\textbf{L}}\}}({\mathbb {R}}^n)={\mathcal {E}}^{\{ {\textbf{L}} \}} ( {\mathbb {R}}^n )\cap {\mathcal {D}}({\mathbb {R}}^n)$, there are constants $C_0$ and $h_0$ such that for $\nu \in {\mathbb {N}}_0^n$

4.9 $$\begin{aligned} {|} D^\nu \psi (y) {|}\le C_0h_0^{|\nu |}L_{|\nu |} \end{aligned}$$

for all $y\in {\mathbb {R}}^n$. We may assume that $h_0\ge 2C_P$.

We prove the statement by induction on *k* where the induction hypothesis for step *k* is that (4.8) is satisfied for all $\nu$, *x* and *t*. For $k=0$ it is clear that (4.8) follows from (4.9). Assuming that (4.8) is true at step *k* we will show that (4.8) is still true at step $k+1$ if we choose the constants suitable.

For simplicity, we set

$$\begin{aligned} \rho&=t^{1-\varepsilon /d},\\ R&=t^{\varepsilon (2-1/d)}. \end{aligned}$$

Observe that $\rho ^d=t^{d-\varepsilon }$ and that according to Lemma 3.1 we have that

$$\begin{aligned} \rho ^d L_{|\nu |+dk} R^{dk}\le \rho ^{d(k+1)}L_{|\nu |}+L_{|\nu |+d(k+1)}R^{d(k+1)}. \end{aligned}$$

If we furthermore put

$$\begin{aligned} \Lambda (k,\nu )= \rho ^{dk}L_{|\nu |}+R^{dk}L_{|\nu |+dk} \end{aligned}$$

then we conclude that

4.10 $$\begin{aligned} \begin{aligned} t^{d-\varepsilon }\Lambda (k,\nu )&= \rho ^{d(k+1)}L_{|\nu |}+\rho ^dL_{|\nu |+dk}R^{dk}\\&\le 2\Lambda (k+1,\nu ) \end{aligned} \end{aligned}$$

and for all ${|\alpha |}\le d$, applying again Lemma 3.1, we obtain

4.11 $$\begin{aligned} \begin{aligned} \rho ^{d-{|\alpha |}}R^{{|\alpha |}}\Lambda (k,\nu +\alpha )&= \rho ^{d(k+1)-{|\alpha |}}L_{|\nu |+{|\alpha |}}R^{|\alpha |}+\rho ^{d-{|\alpha |}}L_{|\nu |+{|\alpha |}+dk}R^{{|\alpha |}+dk}\\&\le 2\rho ^{d(k+1)}L_{|\nu |}+2R^{d(k+1)}L_{|\nu |+d(k+1)}\\&= 2\Lambda (k+1,\nu ). \end{aligned} \end{aligned}$$

We continue by differentiating (4.7b):

$$\begin{aligned} D_x^\nu Q_{k+1}(x,t)=\sum _{{|\alpha |}\le d}\sum _{\nu ^\prime \le \nu }\frac{1}{\alpha !}\left( {\begin{array}{c}\nu \\ \nu ^\prime \end{array}}\right) \left( D_x^{\nu -\nu ^\prime }\partial ^\alpha _{\xi }p\right) (x,t\xi _0)D^{\nu ^\prime +\alpha }_xQ_k(x,t). \end{aligned}$$

We estimate ${|} D_x^\nu Q_{k+1}(x,t) {|}$ by $I_1$, $I_2$ and $I_3$ where:

$$\begin{aligned} I_1&={|} p(x,t\xi _0) {|}{|} D_x^\nu Q_k (x,t) {|}\\ I_2&=\sum _{\nu ^\prime<\nu }\left( {\begin{array}{c}\nu \\ \nu ^\prime \end{array}}\right) {|} D_x^{\nu -\nu ^\prime } p(x,t\xi _0) {|}{|} D_x^{\nu ^\prime } Q_k(x,t) {|}\\ I_3&=\sum _{0<{|\alpha |}\le d}\sum _{\nu ^\prime \le \nu }\frac{1}{\alpha !}\left( {\begin{array}{c}\nu \\ \nu ^\prime \end{array}}\right) {|} D_x^{\nu -\nu ^\prime }\partial _\xi ^\alpha p(x,t\xi _0) {|} {|} D_x^{\nu ^\prime +\alpha } Q_k(x,t) {|} \end{aligned}$$

Now observe that if *t* is fixed the support of $Q_k(.,t)$ is contained in the set $B_t=\left\{ x:{|} x-x_0 {|}\le 2\delta t^{-\varepsilon } \right\}$ for all *k*. Thence by utilizing the induction hypothesis and (4.1) we obtain that

$$\begin{aligned} I_1&\le Dt^{d-\varepsilon }C_0\left( h_0t^\varepsilon \right) ^{|\nu |}\Lambda (k,\nu ) A^k \end{aligned}$$

and thus (4.10) implies that

4.12 $$\begin{aligned} I_1&\le C_0\left( h_0t^\varepsilon \right) ^{|\nu |}2D\Lambda (k+1,\nu )A^k. \end{aligned}$$

Similarly, by (4.5) and the induction hypothesis we have that

$$\begin{aligned} I_2 \le A^k\sum _{\nu ^\prime <\nu }\left( {\begin{array}{c}|\nu |\\ |\nu ^\prime |\end{array}}\right) C_P^{{|} \nu -\nu ^\prime {|} +1}t^d L_{{|} \nu -\nu ^\prime {|}} C_0\left( h_0t^\varepsilon \right) ^{|\nu ^\prime |}\Lambda \left( k,\nu ^\prime \right) . \end{aligned}$$

By also writing $t^d=t^\varepsilon t^{d-\varepsilon }$ and again using (4.10) we obtain

$$\begin{aligned} I_2\le A^k\sum _{\nu ^\prime <\nu }\left( {\begin{array}{c}|\nu |\\ |\nu ^\prime |\end{array}}\right) C_ P^{{|} \nu -\nu ^\prime {|}+1}t^\varepsilon L_{{|} \nu -\nu ^\prime {|}} C_0 \left( h_0t^\varepsilon \right) ^{|\nu ^\prime |}2\Lambda \left( k+1,\nu ^\prime \right) . \end{aligned}$$

According to Lemma 3.2 we have the following estimate

4.13 $$\begin{aligned} \left( {\begin{array}{c}|\nu |\\ |\nu ^\prime |\end{array}}\right) L_{{|} \nu -\nu ^\prime {|}}\Lambda \left( k+1,\nu ^\prime \right) \le \Lambda (k+1,\nu ). \end{aligned}$$

Thence

$$\begin{aligned} I_2\le \sum _{\nu ^\prime <\nu } \left( \frac{C_P}{h_0t^\varepsilon }\right) ^{{|} \nu -\nu ^\prime {|}} t^\varepsilon C_0C_P \left( h_0t^\varepsilon \right) ^{|\nu |}2\Lambda (k+1,\nu )A^k. \end{aligned}$$

If we recall that we have chosen $h_0\ge 2C_P$ and $t\ge 1$ and set

$$\begin{aligned} E=\sum _{\alpha \ge 0}\frac{1}{2^{|\alpha |}} \end{aligned}$$

then

4.14 $$\begin{aligned} I_2 \le \frac{2C_P}{h_0}EC_0C_P\left( h_0t^\varepsilon \right) ^{|\nu |}\Lambda (k+1,\nu ) A^k. \end{aligned}$$

Finally, according to (4.5) and the induction hypothesis we can estimate $I_3$ by

$$\begin{aligned} I_3\le \sum _{0<{|\alpha |}\le d}\sum _{\nu ^\prime \le \nu }\left( {\begin{array}{c}|\nu |\\ |\nu ^\prime |\end{array}}\right) C_P^{{|} \nu -\nu ^\prime {|}+1}L_{{|} \nu -\nu ^\prime {|}}t^{d-{|\alpha |}}C_0\left( h_0t^\varepsilon \right) ^{|\nu ^\prime |+{|\alpha |}} \Lambda (k,\nu ^\prime +\alpha )A^k. \end{aligned}$$

By the definition of $\rho$ and *R* we have for all $\alpha \ne 0$ that

$$\begin{aligned} t^{d-{|\alpha |}+\varepsilon {|\alpha |}}\le \rho ^{d-{|\alpha |}}R^{{|\alpha |}} \end{aligned}$$

which together with (4.11) implies that

$$\begin{aligned} t^{d-{|\alpha |}+\varepsilon {|\alpha |}}\Lambda \left( k,\nu ^\prime +\alpha \right) \le 2\Lambda \left( k+1,\nu ^\prime \right) . \end{aligned}$$

Thus with (4.13) we obtain that

$$\begin{aligned} I_3\le \sum _{0<{|\alpha |}\le d}\sum _{\nu ^\prime \le \nu } \left( \frac{C_P}{h_0t^\varepsilon }\right) ^{{|} \nu -\nu ^\prime {|}} C_PC_0h_0^{|\alpha |}\left( h_0t^\varepsilon \right) ^{|\nu |}2\Lambda (k+1,\nu )A^k. \end{aligned}$$

Setting $h_1=\sum _{{|\alpha |}\le d}h_0^{|\alpha |}$ it follows that

4.15 $$\begin{aligned} I_3\le 2Eh_1C_PC_0\left( h_0t^\varepsilon \right) ^{|\nu |}\Lambda (k+1,\nu )A^{k}. \end{aligned}$$

Combining (4.12), (4.14) and (4.15) we see that

$$\begin{aligned} I_1+I_2+I_3\le C_0\left( h_0t^\varepsilon \right) ^{|\nu |}\Lambda (k+1,\nu )A^{k+1} \end{aligned}$$

if we choose

$$\begin{aligned} A\ge 2D+\frac{2C_PE}{h_0}+2Eh_1C_P. \end{aligned}$$

$$\square $$

If we set $\nu =0$ in (4.8) we obtain that

4.16 $$\begin{aligned} {|} Q_k(x,t) {|}\le C_0A^k\left( \rho ^{dk}+R^{dk}L_{dk}\right) \end{aligned}$$

where we recall that $\rho =t^{1-\varepsilon /d}$ and $R=t^{\varepsilon (2-1/d)}$. By the definition of $\omega _{\textbf{M}}$ we have that

$$\begin{aligned} \rho ^{dk}\le M_{dk}\exp \left( \omega _{\textbf{M}}(\rho )\right) . \end{aligned}$$

The second term on the right-hand side of (4.16) must be dealt with differently. If we assume that there is a weight sequence ${\textbf{V}}$ such that ${\textbf{L}}{\textbf{V}}\preceq {\textbf{M}}$ then using

$$\begin{aligned} R^{dk}\le V_{dk}\exp \left( \omega _{{\textbf{V}}}(R)\right) , \end{aligned}$$

we conclude that there are constants $C, h>0$ such that

$$\begin{aligned} {|} Q_k(x,t) {|}\le Ch^k M_{dk}\left( \exp \left( \omega _{\textbf{M}}\left( \rho \right) \right) + \exp \left( \omega _{\textbf{V}}\left( R\right) \right) \right) . \end{aligned}$$

Now we recall from (3.8) that there are constants $A_2,B_2\ge 1$ such that

$$\begin{aligned} {|} \Phi _{\textbf{N}}(t) {|}\le A_2\exp \left( -\omega _{\textbf{N}}\left( \frac{t}{B_2}\right) \right) \end{aligned}$$

for $t>0$. Combining these estimates we obtain from (4.6) that

4.17 $$\begin{aligned} {|} P^ku(x) {|}\le &   A_2Ch^k M_{dk} \int _1^\infty \negthickspace \exp \left( -\omega _{\textbf{N}}\left( \frac{t}{B_2}\right) \right) \Biggl (\exp \left( \omega _{\textbf{M}}\left( t^{1-\varepsilon /d}\right) \right) \nonumber \\    &   + \exp \left( \omega _{\textbf{V}}\left( t^{\varepsilon (2-1/d)}\right) \right) \Biggr )\,dt \end{aligned}$$

for all $x\in B_0$ and all $k\in {\mathbb {N}}_0$. If we could choose ${\textbf{N}}$, ${\textbf{V}}$ and $\varepsilon$ in such a way that the integral in (4.17) converges then we would have shown that $u\in {\mathcal {E}}^{\{ {\textbf{M}} \}} \left( \Omega ; P \right)$, since ${{\,\textrm{supp}\,}}u\subseteq B_{0}$.

As a first abstract result we note:

### Theorem 4.3

Let ${\textbf{L}}$, ${\textbf{M}}$, ${\textbf{N}}$, ${\textbf{V}}$ be four weight sequences which satisfy the following properties for some $d\in {\mathbb {N}}$:

- $${\textbf{M}}\precnapprox {\textbf{N}}$$ and $$\gamma ({\textbf{N}})>0$$.
- ${\textbf{L}}$ is strongly non-quasianalytic.
- $${\textbf{V}}\le {\textbf{M}}$$ and $${\textbf{L}}{\textbf{V}}\preceq {\textbf{M}}$$.
- There are constants $1<\tau <2d/(2d-1)$ and $A>1$ such that ${\textbf{N}}\le A{\textbf{V}}^{\tau }$.

If *P* is a non-elliptic linear differential operator of order *d* with coefficients in ${\mathcal {E}}^{\{ {\textbf{L}} \}} ( \Omega )$, then there is a smooth function $u\in {\mathcal {E}}(\Omega )$ such that $u\in {\mathcal {E}}^{\{ {\textbf{M}} \}} \left( \Omega ; P \right)$ and $u\notin {\mathcal {E}}^{\{ {\textbf{T}} \}} ( \Omega )$ for any weight sequence ${\textbf{T}}\precnapprox {\textbf{N}}$. Thus in particular $u\notin {\mathcal {E}}^{\{ {\textbf{M}} \}} ( \Omega )$.

### Proof

There is an optimal $\{{\textbf{N}}\}$-flat function $G_{\textbf{N}}$ since $\gamma ({\textbf{N}})>0$. Thus we can define the function *u* by (4.3), i.e.

$$\begin{aligned} u(x)=\int _{1}^{\infty }\negthickspace \psi \bigl (t^\varepsilon (x-x_0)\bigr )\Phi _{\textbf{N}}(t) e^{it\xi _0(x-x_0)}\,dt \end{aligned}$$

where $\Phi _{\textbf{N}}(t)=G_{\textbf{N}}(1/t)$, $(x_0,\xi _0)$ is a non-elliptic point of *P*, $\psi \in {\mathcal {D}}_{\{{\textbf{L}}\}}({\mathbb {R}}^n)$ is a function with suitable compact support as above and $0<\varepsilon <1$ is a parameter to be determined. The estimate (4.4) gives that $u\notin {\mathcal {E}}^{\{ {\textbf{T}} \}} ( \Omega )$ for any weight sequence ${\textbf{T}}\precnapprox {\textbf{N}}$.

In order to estimate the iterates $P^ku$ we apply Lemma 4.2 and since ${\textbf{L}}{\textbf{V}}\preceq {\textbf{M}}$ we obtain (4.17). If $\varepsilon \le 1/2$ then $t^{\varepsilon (2-1/d)}\le t^{1-\varepsilon /d}$ for all $t\ge 1$. Hence $\omega _{\textbf{V}}(t^{\varepsilon (2-1/d)})\le \omega _{\textbf{V}}(t^{1-\varepsilon /d})$. On the other hand, due to ${\textbf{V}}\le {\textbf{M}}$ we have $\omega _{\textbf{M}}(s)\le \omega _{\textbf{V}}(s)$ for all $s\ge 0$. Thus, in summary there are constants $C,h>0$ and a constant $B_2>1$ such that

$$\begin{aligned} {|} P^ku(x) {|}\le Ch^kM_{dk} \int _1^\infty \negthickspace \exp \left( -\omega _{\textbf{N}}\left( \frac{t}{B_2}\right) +\omega _{\textbf{V}}\left( t^{1-\varepsilon /d}\right) \right) \,dt. \end{aligned}$$

By assumption there are $A\ge 1$ and $1<\tau <2d/(2d-1)$ such that ${\textbf{N}}\le A{\textbf{V}}^\tau$. We choose $\varepsilon \le 1/2$ such that

$$\begin{aligned} \tau<\frac{d}{d-\varepsilon }<\frac{2d}{2d-1}. \end{aligned}$$

Thus we are able to apply Lemma 3.7 and according to (3.5) there is a constant ${\widetilde{C}}>0$ such that

$$\begin{aligned} \omega _{\textbf{V}}(s)\le \tau ^{-1}\omega _{\textbf{N}}\left( \frac{s^{d/(d-\varepsilon )}}{B_2}\right) +{\widetilde{C}} \end{aligned}$$

for all $s\ge 0$. If we set $s=t^{1-\varepsilon /d}$ then we obtain

$$\begin{aligned} -\omega _{\textbf{N}}\left( \frac{t}{B_2}\right) +\omega _{\textbf{V}}\left( t^{1-\varepsilon /d}\right) \le -\left( 1-\tau ^{-1}\right) \omega _{\textbf{N}}\left( \frac{t}{B_2}\right) +{\widetilde{C}}. \end{aligned}$$

It follows that there are constant $C,h>0$ such that

$$\begin{aligned} \Vert {P^ku}\Vert _{L^2(B_{0})}\le Ch^k M_{dk} \int _1^\infty \negthickspace \exp \left( -\bigl (1-\tau ^{-1}\bigr )\omega _{\textbf{N}}\bigl (t/B_2\bigr ) \right) \,dt. \end{aligned}$$

The integral on the right-hand side of the above estimate converges since $1-\tau ^{-1}>0$ and $\omega _{\textbf{N}}(s)$ increases faster then $\log s^p$ for any $p\in {\mathbb {N}}$ when $s\rightarrow \infty$, see [29]. Therefore $u\in {\mathcal {E}}^{\{ {\textbf{M}} \}} \left( \Omega ; P \right)$ because ${{\,\textrm{supp}\,}}u\subseteq B_{0}$. $\square$

### Corollary 4.4

Let ${\textbf{M}}$ be a weight sequence such that $\gamma ({\textbf{M}})=\infty$ and ${\textbf{T}}$ be a weight sequence satisfying ${\textbf{T}}\preceq {\textbf{M}}^\rho$ for all $\rho >0$. If *P* is a non-elliptic differential operator with coefficients in ${\mathcal {E}}^{\{ {\textbf{T}} \}} ( \Omega )$ then there is a smooth function *u* such that

$$\begin{aligned} u\in {\mathcal {E}}^{ ({\textbf{M}}) } ( \Omega ; P )\!\setminus \!{\mathcal {E}}^{\{ {\textbf{M}} \}} ( \Omega ). \end{aligned}$$

### Proof

Let *d* be the order of *P*. We choose real parameters $0<q,\sigma <1$ and $\rho >1$ such that

$$\begin{aligned} 1<\rho<\frac{2d}{2d-1}\sigma \quad \text {and}\quad 1<\rho q. \end{aligned}$$

We set ${\widetilde{{\textbf{M}}}}={\textbf{M}}^q$, ${\textbf{L}}={\widetilde{{\textbf{M}}}}^{1-\sigma }={\textbf{M}}^{q(1-\sigma )}$, ${\textbf{V}}={\widetilde{{\textbf{M}}}}^{\sigma }={\textbf{M}}^{q\sigma }$ and ${\textbf{N}}={\widetilde{{\textbf{M}}}}^{\rho }={\textbf{M}}^{q\rho }$. Then ${\textbf{T}}\preceq {\textbf{L}}\lhd {\widetilde{{\textbf{M}}}}\lhd {\textbf{M}}\lhd {\textbf{N}}$. Moreover $\gamma ({\textbf{L}})=\infty$ and thus ${\textbf{L}}$ is strongly non-quasianalytic.

Finally ${\textbf{V}}\le {\widetilde{{\textbf{M}}}}$ and obviously ${\textbf{L}}{\textbf{V}}\preceq {\widetilde{{\textbf{M}}}}$. It follows also that $\gamma ({\textbf{N}})>0$ and ${\textbf{N}}={\textbf{V}}^{\tau }$ where $\tau =\rho /\sigma$.

Hence we can apply Theorem 4.3 and infer the existence of a function $u\in {\mathcal {E}}(\Omega )$ such that $u\in {\mathcal {E}}^{\{ {\widetilde{{\textbf{M}}}} \}} \left( \Omega ; P \right) \subseteq {\mathcal {E}}^{ ({\textbf{M}}) } ( \Omega ; P )$ and $u\notin {\mathcal {E}}^{\{ {\textbf{M}} \}} ( \Omega )$ since ${\widetilde{{\textbf{M}}}}\lhd {\textbf{M}}\lhd {\textbf{N}}$. $\square$

Observe that the proof of Corollary 4.4 works also for non-elliptic differential operators *P* with coefficients of class $\{{\textbf{M}}^\tau \}$ when $\tau$, depending on the order of *P*, is small enough.

In order to finish the proof of Theorem 2.3 it remains to consider the case when $\gamma ({\textbf{M}})>1$ is finite. For this it turns out to be convenient to follow the original proof of Métivier in the Gevrey case more closely.

### Theorem 4.5

Let ${\textbf{M}}$ be a weight sequence such that $1<\gamma ({\textbf{M}})<\infty$. If *P* is a non-elliptic differential operator of class $\{{\textbf{M}}^\rho \}$, for some $1<1/\rho <\gamma ({\textbf{M}})$, in $\Omega$ then there is a smooth function $u\in {\mathcal {E}}(\Omega )$ such that

$$\begin{aligned} u\in {\mathcal {E}}^{ ({\textbf{M}}) } ( \Omega ; P )\!\setminus \!{\mathcal {E}}^{\{ {\textbf{M}} \}} ( \Omega ). \end{aligned}$$

### Proof

We define a new weight sequence ${\textbf{T}}$ by setting

$$\begin{aligned} T_k=M_k^{1/\gamma },\quad k\in {\mathbb {N}}_0, \end{aligned}$$

where $\gamma =\gamma ({\textbf{M}})$. Note that $\gamma ({\textbf{T}})=1$. We can define a scale, i.e. a one-parameter family, of weight sequences by ${\textbf{T}}^\sigma$, $\sigma >1$. Then ${\textbf{T}}^\gamma ={\textbf{M}}$ and ${\textbf{T}}^{\rho \gamma }={\textbf{M}}^\rho$. We will denote the weight function associated to ${\textbf{T}}^\sigma$ by $\omega _\sigma$ and the weight function associated to ${\textbf{T}}={\textbf{T}}^1$ by $\omega =\omega _1$. Clearly

4.18 $$\begin{aligned} \omega _\sigma (s)=\sigma \omega \bigl (s^{1/\sigma }\bigr ) \end{aligned}$$

for all $s\ge 0$. Now choose positive numbers $\gamma _0$ and ${\widetilde{\gamma }}$ such that $\rho \gamma<\gamma _0<{\widetilde{\gamma }}<\gamma$ and

$$\begin{aligned} \frac{\gamma -\gamma _0}{2d}>\gamma -{\widetilde{\gamma }}, \end{aligned}$$

where *d* denotes the order of *P*. Thus ${\textbf{T}}^{\gamma _0}$ is a strongly non-quasianalytic weight sequence since $\gamma ({\textbf{T}}^{\gamma _0})=\gamma _0>\rho \gamma >1$.

Obviously ${\textbf{T}}^{\gamma _0}{\textbf{T}}^{{\widetilde{\gamma }}-\gamma _0}= {\textbf{T}}^{{\widetilde{\gamma }}}$ and we may write ${\textbf{L}}={\textbf{T}}^{\gamma _0}$, ${\textbf{V}}={\textbf{T}}^{{\widetilde{\gamma }}-\gamma _0}$ and ${\widetilde{{\textbf{M}}}}={\textbf{T}}^{{\widetilde{\gamma }}}$. We set also

$$\begin{aligned} \varepsilon&=\frac{d({\widetilde{\gamma }}-\gamma _0)}{2d{\widetilde{\gamma }}-\gamma _0}<\frac{1}{2},\\ \gamma ^\prime&=\frac{d{\widetilde{\gamma }}}{d-\varepsilon }=\frac{2d{\widetilde{\gamma }}-\gamma _0}{2d-1}. \end{aligned}$$

Observe that $\gamma <\gamma ^\prime$. We put ${\textbf{N}}={\textbf{T}}^{\gamma ^\prime }$ and note that ${\widetilde{{\textbf{M}}}}\lhd {\textbf{M}}\lhd {\textbf{N}}$. Since $\gamma ({\textbf{N}})=\gamma ^\prime >0$ we can define *u* by (4.3) using the above choices of ${\textbf{L}}$, ${\textbf{N}}$ and $\varepsilon$. Then it follows from (4.4) that *u* cannot be of class $\{{\textbf{M}}\}$ in $\Omega$ since ${\textbf{M}}\lhd {\textbf{N}}$.

We recall that

$$\begin{aligned} P^ku(x)=\int _1^\infty \negthickspace Q_k(x,t)\Phi _{{\textbf{N}}}(t)e^{it\xi _0(x-x_0)}\,dt \end{aligned}$$

where the functions $Q_k$ are defined by (4.7). If we want to estimate ${|} P^ku(x) {|}$ then we observe that

4.19 $$\begin{aligned} {|} P^ku(x) {|}\le C_2\int _1^\infty \negthickspace {|} Q_k(x,t) {|}e^{-\omega _{\textbf{N}}(t/B_2)}\,dt \end{aligned}$$

for some constants $C_2>0$ and $B_2>1$. According to Lemma 4.2 there are constants $C,h>0$ such that

$$\begin{aligned} {|} Q_k(x,t) {|}\le Ch^k\left( t^{(d-\varepsilon )k}+t^{k\varepsilon (2d-1)}L_{dk}\right) . \end{aligned}$$

If we set

$$\begin{aligned} \rho&=t^{1-\varepsilon /d}  &   \text {and}&R&=t^{\varepsilon (2-1/d)} \end{aligned}$$

then

$$\begin{aligned} \rho ^{dk}&=t^{(d-\varepsilon )k}  &   \text {and}&R^{dk}&=t^{k\varepsilon (2d-1)}. \end{aligned}$$

Observe now that

$$\begin{aligned} \frac{\rho ^{dk}}{q^{dk}}&\le {\widetilde{M}}_{dk}\exp \left( \omega _{{\widetilde{{\textbf{M}}}}}\left( \frac{\rho }{q}\right) \right) \end{aligned}$$

and

$$\begin{aligned} \frac{R^{dk}}{q_1^{dk}}&\le V_{dk} \exp \left( \omega _{{\textbf{V}}}\left( \frac{R}{q_1}\right) \right) \end{aligned}$$

for any $q,q_1>0$. We set $q=B_2^{(d-\varepsilon )/d}$ and $q_1=B_2^{\varepsilon (2d-1)/d}$ where $B_2$ is the constant in the exponential in (4.19). We have $q>q_1$ since $\varepsilon <1/2$ and $B_2>1$. Moreover we infer from (4.18) that

$$\begin{aligned} \omega _{{\widetilde{{\textbf{M}}}}}(s)&={\widetilde{\gamma }}\omega \left( s^{1/{\widetilde{\gamma }}}\right) ,\\ \omega _{\textbf{V}}(s)&=\gamma _1\omega \left( s^{1/\gamma _1}\right) ,\\ \omega _{\textbf{N}}(s)&=\gamma ^\prime \omega \left( s^{1/\gamma ^\prime }\right) \end{aligned}$$

for $s\ge 0$. Here $\omega$ is the associated weight function to ${\textbf{T}}={\textbf{M}}^{1/\gamma }$ and $\gamma _1={\widetilde{\gamma }}-\gamma _0$. In particular

$$\begin{aligned} \omega _{{\widetilde{{\textbf{M}}}}}\left( \frac{\rho }{q}\right)&={\widetilde{\gamma }} \omega \left( \frac{\left( t^{(d-\varepsilon )/d}\right) ^{1/{\widetilde{\gamma }}}}{ \left( B_2^{(d-\varepsilon )/d}\right) ^{1/{\widetilde{\gamma }}}}\right) ={\widetilde{\gamma }}\omega \left( \frac{t^{1/\gamma ^\prime }}{B_2^{1/\gamma ^\prime }}\right) ,\\ \omega _{\textbf{V}}\left( \frac{R}{q_1}\right)&=\gamma _1\omega \left( \frac{\left( t^{\varepsilon (2d-1)/d}\right) ^{1/\gamma _1}}{\left( B_2^{\varepsilon (2d-1)/d}\right) ^{1/ \gamma _1}}\right) =\gamma _1\omega \left( \frac{t^{1/\gamma ^\prime }}{B_2^{1/\gamma ^\prime }}\right) \end{aligned}$$

since $$\gamma _1d/(\varepsilon (2d-1))=\gamma ^\prime $$. Finally

$$\begin{aligned} \omega _{\textbf{N}}\left( \frac{t}{B_2}\right)&=\gamma ^\prime \omega \left( \frac{t^{1/\gamma ^\prime }}{B_2^{1/\gamma ^\prime }}\right) . \end{aligned}$$

Thus we can conclude that $\omega _{\textbf{V}}(R/q_1)\le \omega _{{\widetilde{{\textbf{M}}}}}(\rho /q)$ since $\gamma _1<{\tilde{\gamma }}$. Therefore we have the following estimate:

$$\begin{aligned} {|} Q_k(x,t) {|}\le 2C \left( q^dh\right) ^k {\widetilde{M}}_{dk}\exp \left( \omega _{{\widetilde{{\textbf{M}}}}}\left( \frac{\rho }{q}\right) \right) . \end{aligned}$$

It follows that

4.20 $$\begin{aligned} {|} P^ku(x) {|}\le 2CC_2\left( q^dh\right) ^k{\widetilde{M}}_{dk}\int _1^\infty \negthickspace \exp \left( -\omega _{\textbf{N}}\left( \frac{t}{B_2}\right) +\omega _{{\widetilde{{\textbf{M}}}}}\left( \frac{\rho }{q}\right) \right) \,dt. \end{aligned}$$

However, our arguments above infer that

$$\begin{aligned} -\omega _{\textbf{N}}\left( \frac{t}{B_2}\right) +\omega _{{\widetilde{{\textbf{M}}}}}\left( \frac{\rho }{q}\right) =-\gamma ^\prime \omega \left( \frac{t^{1/\gamma ^\prime }}{B_2^{1/\gamma ^\prime }}\right) +{\widetilde{\gamma }}\omega \left( \frac{t^{1/\gamma ^\prime }}{B_2^{1/\gamma ^\prime }}\right) =-\bigl (\gamma ^\prime -{\tilde{\gamma }}\bigr ) \omega \left( \frac{t^{1/\gamma ^\prime }}{B_2^{1/\gamma ^\prime }}\right) . \end{aligned}$$

Since $\gamma ^\prime >{\widetilde{\gamma }}$ and the associated weight function $\omega (s)$ grows faster than any $\log s^p$, $p\in {\mathbb {N}}$, we conclude that the integral in (4.20) converges and therefore $u\in {\mathcal {E}}^{\{ {\widetilde{{\textbf{M}}}} \}} \left( \Omega ; P \right)$ because ${{\,\textrm{supp}\,}}u\subseteq B_{0}$. Since ${\widetilde{{\textbf{M}}}}\lhd {\textbf{M}}$ we obtain that $u\in {\mathcal {E}}^{ ({\textbf{M}}) } ( \Omega ; P )$. $\square$

If we apply Theorem 4.5 to the Gevrey case we obtain the following Corollary.

### Corollary 4.6

Let $1\le r<s$ and $\Omega \subseteq {\mathbb {R}}^n$ be an open set. If *P* is a non-elliptic differential operator with coefficients in ${\mathcal {G}}^r(\Omega )$ then there is a smooth function $u\in {\mathcal {E}}(\Omega )$ such that

$$\begin{aligned} u\in {\mathcal {G}}^s(\Omega ;P)\!\setminus \!{\mathcal {G}}^s(\Omega ). \end{aligned}$$

### Proof

Since the case $r=1$ is just [32, Theorem 2.3], we can assume that $r>1$. If we put ${\textbf{M}}={\textbf{G}}^s$ then ${\textbf{G}}^r={\textbf{M}}^{\rho }$ with $\rho =r/s$. In particular $1<1/ \rho < s=\gamma ({\textbf{M}})$. Hence the assertion follows from Theorem 4.5. $\square$

Combining Corollary 4.6 with [5, Theorem 1.1] gives immediately Theorem 2.9.

## Optimal functions in Denjoy–Carleman classes

### Definition 5.1

Let ${\textbf{N}}$ be a weight sequence. We say that a smooth function $g\in {\mathcal {E}}(\Omega )$ on an open set $\Omega \subseteq {\mathbb {R}}^n$ is an optimal function of the Roumieu class ${\mathcal {E}}^{\{ {\textbf{N}} \}} ( \Omega )$ if $g\in {\mathcal {E}}^{\{ {\textbf{N}} \}} ( \Omega )$ but $g\notin {\mathcal {E}}^{\{ {\textbf{T}} \}} ( \Omega )$ for any weight sequence ${\textbf{T}}\precnapprox {\textbf{N}}$.

We can use the general approach to the construction of the function *u* in Sect. 4 to define optimal functions in Denjoy–Carleman classes. Indeed, assume that ${\textbf{N}}$ is a weight sequence which satisfies (2.6) and $\gamma ({\textbf{N}})>0$. Then there exists an optimal $\{{\textbf{N}}\}$-flat function $G_{\textbf{N}}$ which is defined in some sector $S_\gamma$, $\gamma <\gamma ({\textbf{N}})$. Now let $x_0\in {\mathbb {R}}^n$ and $\xi _0\in S^{n-1}$. If we set

$$\begin{aligned} g(x)=\int _0^\infty \negthickspace \Phi _{\textbf{N}}(t)e^{it\xi _0(x-x_0)}\,dt, \end{aligned}$$

where $\Phi _{\textbf{N}}(s)=G_{\textbf{N}}(1/s)$, then *g* is a smooth function defined on ${\mathbb {R}}^n$. We see as before that

$$\begin{aligned} D_{\xi _0}^kg(x_0)=\int _0^\infty \negthickspace t^k\Phi _{\textbf{N}}(t)\,dt \end{aligned}$$

and thence, according to Proposition 3.9,

$$\begin{aligned} D_{\xi _0}^kg(x_0)\ge B_1^{k+1}N_k \end{aligned}$$

for some constant $B_1>0$. Hence *g* cannot be of class $\{{\textbf{T}}\}$ near $x_0$ for any weight sequence ${\textbf{T}}\precnapprox {\textbf{N}}$. On the other hand we can show that *g* is of class $\{{\textbf{N}}\}$ in $\Omega$ if ${\textbf{N}}$ satisfies (2.6). Indeed we have that

$$\begin{aligned} {|} D^\alpha g(x) {|}\le \int _0^\infty \negthickspace t^{|\alpha |}\Phi _{\textbf{N}}(t)\,dt \le CQ_2^{|\alpha |}N_{|\alpha |}\end{aligned}$$

for some constants $C,Q_2>0$ by Proposition 3.9. Hence *g* is an optimal function in the class ${\mathcal {E}}^{\{ {\textbf{N}} \}} ( {\mathbb {R}}^n )$.

We may point out that this approach defining optimal functions in Denjoy–Carleman classes is more flexible than the usual one by Fourier series, see e.g. [37]. For example, we can specify not only the point but also the direction where the optimality occurs. Depending on the problem, one can further modify the construction of optimal functions. Indeed, we can show, for example, that the function *u* from Theorem 2.3, if the weight sequence ${\textbf{M}}$ satisfies (2.6), is an optimal function in a Denjoy–Carleman class ${\mathcal {E}}^{\{ {\textbf{N}} \}} ( \Omega )$ given by a weight sequence ${\textbf{N}}$ depending on ${\textbf{M}}$ and the operator *P*.

In fact, in Sect. 4 the function *u* was constructed in the following way: For a given weight sequence ${\textbf{M}}$ we choose suitable weight sequences ${\textbf{L}}$ and ${\textbf{N}}$ such that ${\textbf{L}}\preceq {\textbf{M}}\precnapprox {\textbf{N}}$, ${\textbf{L}}$ is non-quasianalytic and $\gamma ({\textbf{N}})>0$. Then we define

$$\begin{aligned} u(x)=\int _1^\infty \negthickspace \psi (t^\varepsilon (x-x_0))\Phi _{\textbf{N}}(t)e^{it\xi _0(x-x_0)}\,dt \end{aligned}$$

where $0<\varepsilon <1$ is some constant, $\xi _0\in S^{n-1}$, $x_0\in \Omega$ and $\psi \in {\mathcal {E}}^{\{ {\textbf{L}} \}} ( {\mathbb {R}}^n )$ is such that $\psi (y)=1$ for ${|} y {|}\le \delta$ and $\psi (y)=0$ for ${|} y {|}\ge 2\delta$ with $\delta >0$ being such that $B_0=\left\{ x\in {\mathbb {R}}^n: {|} x-x_0 {|}\ge 2\delta \right\} \subseteq \Omega$. Furthermore $\Phi _{\textbf{N}}(t)=G_{\textbf{N}}(1/t)$ where $G_{\textbf{N}}$ is an optimal $\{{\textbf{N}}\}$-flat function. From the estimate (4.4) it follows that $u\notin {\mathcal {E}}^{\{ {\textbf{T}} \}} ( \Omega )$ for any weight sequence ${\textbf{T}}\precnapprox {\textbf{N}}$. Here we claim that if ${\textbf{N}}$ satisfies (2.6) then $u\in {\mathcal {E}}^{\{ {\textbf{N}} \}} ( \Omega )$. For this we consider the iterates $D_j^ku$ of *u* with respect to the constant coefficient operator $D_j$, $j\in \left\{ 1,\dotsc ,n \right\}$. We see that

$$\begin{aligned} D_j^ku(x)=\int _1^\infty \negthickspace \Delta _k^j(x,t)\Phi _{\textbf{N}}(t)e^{it\xi _0(x-x_0)}\,dt \end{aligned}$$

where the functions $\Delta _k^j$ are iteratively given by

$$\begin{aligned} \Delta _0^j(x,t)&=\psi (t^\varepsilon (x-x_0)),&j&=1,\dotsc ,n,\\ \Delta _{k+1}^j(x,t)&=D_j\Delta ^j_k(x,t)+t\xi _{0,j}\Delta ^j_k(x,t),&j&=1,\dotsc ,n,\;k\in {\mathbb {N}}_0, \end{aligned}$$

where $\xi _{0,j}$ is the *j*-th component of the vector $\xi _0$. For these functions we have a statement analogous to Lemma 4.2:

### Lemma 5.2

For each $j\in \left\{ 1,\dotsc ,n \right\}$ there is a constant $A_j>0$ such that

5.1 $$\begin{aligned} {|} D^\nu _x\Delta _k^j(x,t) {|}\le C_0(h_0t^\varepsilon )^{|\nu |}A_j^k \left( t^kL_{|\nu |}+t^{\varepsilon k}L_{|\nu |+k}\right) ,\qquad x\in B_0,\;t\ge 1, \end{aligned}$$

for all $\nu \in {\mathbb {N}}_0^n$ and $k\in {\mathbb {N}}_0$. Here $C_0,h_0$ are the constants from (4.9).

### Proof

We will prove the Lemma by induction on $k\in {\mathbb {N}}_0$. In the case $k=0$ we have $\Delta _0^j(x,t)=\psi (t^\varepsilon (x-x_0))$. Thence

$$\begin{aligned} D_x^\nu \left( \psi (t^\varepsilon (x-x_0)\right) =t^{\varepsilon |\nu |}D^\nu \psi (t^\varepsilon (x-x_0)) \end{aligned}$$

and thus (4.9) gives that

$$\begin{aligned} {|} D^\nu _x\Delta _0^j(x,t) {|}\le C_0(h_0t^\varepsilon )^{|\nu |}L_{|\nu |}. \end{aligned}$$

We will now show that if (5.1) holds for *k* then (5.1) is also satisfied for $k+1$ when we choose $A_j$ suitably. For simplicity we set $R=t^\varepsilon$. From Lemma 3.1 we obtain that

$$\begin{aligned} tL_{|\nu |+k}R^k\le t^{k+1}L_{|\nu |}+L_{|\nu |+k+1}R^{k+1}. \end{aligned}$$

We set

$$\begin{aligned} \Theta (k,\nu )=t^kL_{|\nu |}+R^kL_{|\nu |+k} \end{aligned}$$

and conclude that

5.2 $$\begin{aligned} t\Theta (k,\nu )\le t^{k+1}L_{|\nu |}+tL_{|\nu |+k}R^k\le 2\Theta (k+1,\nu ). \end{aligned}$$

For all ${|\alpha |}\le 1$ Lemma 3.1 gives the following estimate:

5.3 $$\begin{aligned} \begin{aligned} t^{1-{|\alpha |}}R^{|\alpha |}\Theta (k,\nu +\alpha )&\le t^{k+1-{|\alpha |}}L_{|\nu |+{|\alpha |}}R^{|\alpha |}+t^{1-{|\alpha |}}L_{|\nu |+{|\alpha |}+k}R^{{|\alpha |}+k}\\&\le 2\left( t^{k+1}L_{|\nu |}+L_{|\nu |+k+1}R^{k+1}\right) \\&=2\Theta (k+1,\nu ). \end{aligned} \end{aligned}$$

Furthermore if $e_j$ denotes the *j*-th unit vector in ${\mathbb {R}}^n$ then we have

$$\begin{aligned} D_x^{\nu }\Delta _{k+1}^j(x,t)=D_x^{\nu +e_j}\Delta _k^j(x,t)+t\xi _{0,j}D_x^\nu \Delta _k^j(x,t). \end{aligned}$$

Thus the induction hypothesis implies that

$$\begin{aligned} \begin{aligned} {|} D_x^\nu \Delta _{k+1}^j(x,t) {|}&\le {|} D_x^{\nu +e_j}\Delta ^j_k(x,t) {|}+{|} t\xi _{0,j}D_x^\nu \Delta ^j_k(x,t) {|}\\&\le C_0(h_0t^\varepsilon )^{|\nu |+1}A_j^k\left( t^kL_{|\nu |+1}+t^{\varepsilon k}L_{|\nu |+k+1}\right) \\&\quad \; +C_0(h_0t^\varepsilon )^{|\nu |}A_j^kt {|} \xi _{0,j} {|}\left( t^{k}L_{|\nu |}+t^{\varepsilon k}L_{|\nu |+k}\right) \\&=C_0(h_0t^\varepsilon )^{|\nu |}A_j^k\left( h_0t^\varepsilon \Theta (k,\nu +e_j)+{|} \xi _{0,j} {|}t\Theta (k,\nu )\right) . \end{aligned} \end{aligned}$$

Now, if we recall that $R=t^\varepsilon$ and $0\le {|} \xi _{0,j} {|}\le 1$ then using (5.2) and (5.3) allows us to conclude that

$$\begin{aligned} {|} D_x^\nu \Delta _{k+1}^j(x,t) {|}\le C_0(h_0t^\varepsilon )^{|\nu |}A_j^k2\left( h_0+ {|} \xi _{0,j} {|}\right) \Theta (k+1,\nu ). \end{aligned}$$

Therefore we have proven the Lemma if we choose $A_j\ge 2(h_0+{|} \xi _{0,j} {|})$. $\square$

### Remark 5.3

We may point out that in the proof of Lemma 5.2 we did not need to use (3.1), contrary to the proof of Lemma 4.2. This is due to the fact that in Lemma 5.2 the differential operators $D_j$ under consideration have constant coefficients.

Moreover, in (5.1) we can replace the constants $A_j$ by a constant *A* independent of *j* by using $A=\max _{j}A_j$.

If we set $\nu =0$ in Lemma 5.2 then

$$\begin{aligned} {|} \Delta _k^j(x,t) {|}\le C_0A_j^k\left( t^k+t^{\varepsilon k}L_k\right) . \end{aligned}$$

Thus we have that

$$\begin{aligned} {|} D_j^k u(x,t) {|}\le C_0A_j^k\left( \int _1^\infty \negthickspace t^k\Phi _{\textbf{N}}(t)\,dt +L_k\int _1^\infty \negthickspace t^{\varepsilon k}\Phi _{\textbf{N}}(t)\,dt\right) \end{aligned}$$

for all $k\in {\mathbb {N}}_0$ and $j=1,\dotsc ,n$. If ${\textbf{N}}$ satisfies (2.6) then by Proposition 3.9 there are constants $C_1,B_1$ such that

$$\begin{aligned} \int _1^\infty \negthickspace t^k\Phi _{\textbf{N}}(t)\,dt\le \int _0^\infty \negthickspace t^k\Phi _{\textbf{N}}(t)\,dt\le C_1B_1^k N_k. \end{aligned}$$

If there were constants $C_2,B_2>0$ such that

5.4 $$\begin{aligned} L_k\int _1^\infty \negthickspace t^{\varepsilon k}\Phi _{\textbf{N}}(t)\,dt\le C_2B_2^k N_k \end{aligned}$$

then we would be able to show that for each $j=1,\dotsc ,n$ there are constants $C_3,h_j>0$ such that

$$\begin{aligned} {|} D_j^k u(x) {|}\le C_3 h_j^k N_k, \end{aligned}$$

i.e. $$u\in {\mathcal {E}}^{\{ {\textbf{N}} \}} \left( \Omega ; D_j \right) $$ for all $$j=1,\dotsc ,n$$.

By our assumptions we know that ${\textbf{N}}$ is non-quasianalytic since ${\textbf{L}}$ is non-quasianalytic. This further implies that ${\textbf{N}}$ satisfies (2.5). Because we suppose here also that (2.6) holds for ${\textbf{N}}$ we know by [7, Corollaire 3] (cf. also [20, Corollary 3.17]) that

$$\begin{aligned} \bigcap _{j=1}^n {\mathcal {E}}^{\{ {\textbf{N}} \}} \left( \Omega ; D_j \right) ={\mathcal {E}}^{\{ {\textbf{N}} \}} ( \Omega ). \end{aligned}$$

Thence $$u\in {\mathcal {E}}^{\{ {\textbf{N}} \}} ( \Omega )$$.

### Theorem 5.4

Assume that the hypothesis of Theorem 4.3 holds. If the weight sequence ${\textbf{N}}$ satisfies additionally (2.6) then the function *u* from Theorem 4.3 is an optimal function of ${\mathcal {E}}^{\{ {\textbf{N}} \}} ( \Omega )$.

### Proof

By assumption we have that there are weight sequences ${\textbf{L}}$, ${\textbf{M}}$, ${\textbf{N}}$, ${\textbf{V}}$ and constants $d\in {\mathbb {N}}$, $A>1$ and $1<\tau < 2d/(2d-1)$ such that $\gamma ({\textbf{N}})>0$, ${\textbf{M}}\preceq {\textbf{N}}$, ${\textbf{L}}$ is non-quasianalytic, ${\textbf{V}}\le {\textbf{M}}$ and ${\textbf{L}}{\textbf{V}}\preceq {\textbf{M}}$. Furthermore ${\textbf{N}}\le A{\textbf{V}}^\tau$ and in the proof of Theorem 4.3 we have chosen $\varepsilon$ such that $\tau<d/(d-\varepsilon )< 2d/(2d-1)$.

Since we suppose also that (2.6) holds for ${\textbf{N}}$ we have only to show (5.4) due to the deliberations above. By (3.8), the definition of $\omega _{\textbf{V}}$ and the fact that ${\textbf{L}}{\textbf{V}}\preceq {\textbf{M}}\precnapprox {\textbf{N}}$ there are constants $C_2,B_2,B>0$ such that

$$\begin{aligned} L_k\int _1^\infty \negthickspace t^{\varepsilon k}\Phi _{\textbf{N}}(t)\,dt\le C_2B_2^k N_k\int _{1}^{\infty }\negthickspace \exp \left( -\omega _{\textbf{N}}\left( \frac{t}{B}\right) +\omega _{\textbf{V}}(t^\varepsilon )\right) \,dt. \end{aligned}$$

Now, since ${\textbf{N}}\le A{\textbf{V}}^{\tau }$ and $\tau<d/(d-\varepsilon )<1/\varepsilon$ for all $d\ge 1$, by our choice above we have that $\varepsilon <1/2$, we can apply Lemma 3.7 and obtain that there is some constant $C>0$ such that

$$\begin{aligned} \omega _{\textbf{V}}(s)\le \tau ^{-1}\omega _{\textbf{N}}\left( \frac{s^{1/\varepsilon }}{B}\right) +C \end{aligned}$$

for all $s\ge 0$. Thence

$$\begin{aligned} \int _1^\infty \negthickspace \exp \left( -\omega _{\textbf{N}}\left( \frac{t}{B}\right) +\omega _{\textbf{V}}(t^\varepsilon )\right) \,dt \le C\int _1^\infty \negthickspace \exp \left( -(1-\tau ^{-1})\omega _{\textbf{N}}\left( \frac{t}{B}\right) \right) \,dt \end{aligned}$$

and the integral on the right-hand side converges since $\tau >1$ and $\omega _{\textbf{N}}(s)$ increases faster than any power of $\log s$. Thus we have proven (5.4) in this case. $\square$

### Corollary 5.5

Suppose that the hypothesis of Corollary 4.4 holds. If the weight sequence ${\textbf{M}}$ additionally satisfies (2.6) then the function *u* in the statement of Corollary 4.4 is an optimal function in ${\mathcal {E}}^{\{ {\textbf{M}}^\nu \}} ( \Omega )$ for some $\nu >1$.

### Proof

Observe that if ${\textbf{M}}$ satisfies (2.6) then (2.6) holds for any weight sequence ${\textbf{M}}^r$, $r>0$. The statement then follows from the proof of Corollary 4.4 if we also take Theorem 5.4 into account. $\square$

### Theorem 5.6

Suppose that the assumptions of Theorem 4.5 hold. If the weight sequence ${\textbf{M}}$ satisfies also (2.6) then there is some $\nu >1$ such that the function *u* in Theorem 4.5 is an optimal function of the class ${\mathcal {E}}^{\{ {\textbf{M}}^\nu \}} ( \Omega )$.

### Proof

By assumption $1<\gamma =\gamma ({\textbf{M}})<\infty$ and as in the proof of Theorem 4.5 we set ${\textbf{T}}={\textbf{M}}^{1/\gamma }$. We use the same choices of $\gamma _0$, $\widetilde{\gamma _0}$, $\gamma ^\prime$ and $\varepsilon$ as in the proof of Theorem 4.5 and set ${\textbf{L}}={\textbf{T}}^{\gamma _0}$, ${\textbf{V}}={\textbf{T}}^{{\tilde{\gamma }}-\gamma _0}$ and ${\textbf{N}}={\textbf{T}}^{\gamma ^\prime }$. Note that ${\textbf{N}}={\textbf{M}}^{\nu }$ with $\nu =\gamma ^\prime /\gamma >1$ since $\gamma ^\prime >\gamma$ and therefore ${\textbf{N}}$ satisfies (2.6) if ${\textbf{M}}$ does.

With these choices of ${\textbf{L}}$, ${\textbf{V}}$, ${\textbf{N}}$ and $\varepsilon$ we have defined *u* in the proof of Theorem 4.5 by (4.3). Thus using the arguments before Theorem 5.4 we have only to check if (5.4) holds in order to show that *u* is an optimal function of the class ${\mathcal {E}}^{\{ {\textbf{N}} \}} ( \Omega )$.

By (3.8) we have that there are constants $A,B>0$ such that

$$\begin{aligned} {|} \Phi _{\textbf{N}}(t) {|}\le A\exp \left( -\omega _{\textbf{N}}\left( \frac{t}{B}\right) \right) \end{aligned}$$

for all $t\ge 0$. Now we use the fact that

$$\begin{aligned} \frac{t^{\varepsilon k}}{B^{\varepsilon k}}\le V_k\exp \left( \omega _{\textbf{V}}\left( \frac{t^\varepsilon }{B^\varepsilon }\right) \right) \end{aligned}$$

to obtain that

5.5 $$\begin{aligned} L_k\int _1^\infty \negthickspace t^{\varepsilon k}\Phi _{\textbf{N}}(t)\,dt\le C_2B_2^k N_k\int _{1}^{\infty }\negthickspace \exp \left( -\omega _{\textbf{N}}\left( \frac{t}{B}\right) + \omega _{\textbf{V}}\left( \frac{t^\varepsilon }{B^\varepsilon }\right) \right) \,dt \end{aligned}$$

for some constants $C_2,B_2>0$ since ${\textbf{V}}\le {\textbf{M}}$ and ${\textbf{L}}{\textbf{V}}\preceq {\textbf{M}}$. If $\omega$ is the weight function associated to ${\textbf{T}}$ then we recall for the weight functions associated to the weight sequences ${\textbf{V}}$ and ${\textbf{N}}$ the following identities from the proof of Theorem 4.5:

$$\begin{aligned} \omega _{{\textbf{N}}}(s)&=\gamma ^\prime \omega \left( s^{1/\gamma ^\prime }\right) ,\qquad s\ge 0,\\ \omega _{{\textbf{V}}}(s)&=\gamma _1\omega \left( s^{1/\gamma _1}\right) , \qquad s\ge 0, \end{aligned}$$

where $\gamma _1={\widetilde{\gamma }}-\gamma _0$. By our choices we have that $\gamma _1/\varepsilon =(2d{\widetilde{\gamma }}-\gamma _0)/d$ and thus $\gamma _1/\varepsilon \ge \gamma ^\prime$. Therefore we conclude that

$$\begin{aligned} \omega _{\textbf{V}}\left( \frac{t^\varepsilon }{B^\varepsilon }\right) =\gamma _1\omega \left( \frac{t^{\varepsilon /\gamma _1}}{B^{\varepsilon /\gamma _1}}\right) \le \gamma _1\omega \left( \frac{t^{1/\gamma ^\prime }}{B^{1/\gamma ^\prime }}\right) \end{aligned}$$

for $t\ge B$ since $\omega$ is an increasing function on $(0,\infty )$. Hence

$$\begin{aligned} -\omega _{{\textbf{N}}}\left( \frac{t}{B}\right) +\omega _{\textbf{V}}\left( \frac{t^\varepsilon }{B^\varepsilon }\right) \le -(\gamma ^\prime -\gamma _1)\omega \left( \frac{t^{1/\gamma ^\prime }}{B^{1/\gamma ^\prime }}\right) \end{aligned}$$

for $t\ge B$ and since $\gamma ^\prime >\gamma _1$ it follows that the integral on the right-hand side of (5.5) converges. Thus we have proven (5.4). $\square$

## Data Availability

Data sharing not applicable to this article as no datasets were generated or analysed during the current study.

## Footnotes

1 An ultradifferentiable structure is in our context a sheaf of smooth functions which is defined by estimates on the derivatives of its elements.

2 A weight function $\omega$ is non-quasianalytic if $\int _1^\infty \omega (t)t^{-2}dt<\infty$, cf. [12]
